# Colloidal Gels with Tunable Mechanomorphology Regulate Endothelial Morphogenesis

**DOI:** 10.1038/s41598-018-37788-w

**Published:** 2019-01-31

**Authors:** Smruti K. Nair, Sukanya Basu, Ballari Sen, Meng-Hsuan Lin, Arati N. Kumar, Yuan Yuan, Paul J. Cullen, Debanjan Sarkar

**Affiliations:** 1 Department of Biomedical Engineering, University at Buffalo, The State University of New York, Buffalo, NY 14260 USA; 2 Department of Biological Sciences, University at Buffalo, The State University of New York, Buffalo, NY 14260 USA; 3 Department of Chemical and Biological Engineering, University at Buffalo, The State University of New York, Buffalo, NY 14260 USA

## Abstract

Endothelial morphogenesis into capillary networks is dependent on the matrix morphology and mechanical properties. In current 3D gels, these two matrix features are interdependent and their distinct roles in endothelial organization are not known. Thus, it is important to decouple these parameters in the matrix design. Colloidal gels can be engineered to regulate the microstructural morphology and mechanics in an independent manner because colloidal gels are formed by the aggregation of particles into a self-similar 3D network. In this work, gelatin based colloidal gels with distinct mechanomorphology were developed by engineering the electrostatic interaction mediated aggregation of particles. By altering the mode of aggregation, colloidal gels showed either compact dense microstructure or tenuous strand-like networks, and the matrix stiffness was controlled independently by varying the particle fraction. Endothelial Cell (EC) networks were favored in tenuous strand-like microstructure through increased cell-matrix and cell-cell interactions, while compact dense microstructure inhibited the networks. For a given microstructure, as the gel stiffness was increased, the extent of EC network was reduced. This result demonstrates that 3D matrix morphology and mechanics provide distinct signals in a bidirectional manner during EC network formation. Colloidal gels can be used to interrogate the angiogenic responses of ECs and can be developed as a biomaterial for vascularization.

## Introduction

Colloidal gels are formed by the aggregation of sub-micron colloidal particles in 3D into interconnected network structures^1–5^. The assembly of these particles through interparticle interactions leads to large-scale gel structure from the organization of particles which are self-similar at certain length scales^3,4,6,7^. Qualitatively, colloidal particles aggregate into clusters, which are hierarchically developed into a self-spanning network to form the gels. The transition of the dispersions of colloidal particles into gels depends on the fraction of particles present in the dispersion and the extent of interparticle interactions, which ultimately define the microstructural organization of particles through a defined mode of particle aggregation^1,8–10^. By varying these two parameters, the mechanical properties and the microstructural morphology of the colloidal gels can be regulated independently. Colloidal gels, therefore, can be used as a 3D matrix to control the functionality of the cells for controlled morphogenesis. In particular, organization of endothelial cells (ECs) into vascular networks is tightly regulated by the stiffness and the morphology of the 3D matrix^11^. ECs require optimal matrix stiffness and the spatial guidance from the matrix to organize into networks. In this context, colloidal gels represent a functional matrix that can provide the necessary microstructural and mechanical cues for endothelial organization. This can allow investigation of the roles of matrix mechanics and morphology in endothelial morphogenesis.

Currently, several 3D hydrogels have been designed to investigate the endothelial morphogenesis where the gels are designed with bioadhesive ligands (e.g. RGD), degradable linkages (e.g. MMP sensitive), and growth factors^12–14^. These physically or chemically crosslinked hydrogels are developed from synthetic and natural polymers e.g. polyethylene glycol^12^, alginates^14^, hyaluronic acid^15^ as well as from reconstituted biological matrices e.g. fibrin^16^, collagen^17^, glycosaminoglycan^18^. However, the exact roles of matrix stiffness and morphology cannot be extracted independently. Specifically, in such gels, these features are intertwined because changing gel density simultaneously alters morphology and mechanics. Moreover, these hydrogels are extremely fibrous with small mesh and provide limited control on the spatial microenvironment of the matrix for cells to organize^19–21^. Within this context, colloidal gels enable the systematic identification of the matrix conditions in terms of its stiffness and morphology, because these gels are formed by the self-similar assembly of particles. Moreover, the assembly of these particles in 3D can regulate the spatial microstructure and guide the cells to organize into functional vascular networks. Thus, by controlling particle aggregation, both matrix mechanics and morphology can be tuned in a controlled manner in a colloidal gel. In this way, vascular network assembly can be studied.

Colloidal gels are extensively researched as materials in terms of their structure-function properties. Interparticle interactions can be achieved by different mechanisms including electrostatic, magnetic, hydrophobic or specific molecular interactions^7,22–27^. By using these interactions along with the particle fraction, the arrangement of colloidal particles can be controlled to develop 3D networks as gel. Mechanistically, colloidal particles can aggregate either by a reaction-limited path to form compact dense clusters, or by a diffusion-limited path to form branched strands; thus, the variation of aggregation modes lead to different degrees of particle organization^28–30^. This allows achieving distinctly different morphologies of the gel and the mechanical strength of the gels increases with increasing particle fraction. Electrostatic interaction mediated aggregation of oppositely charged colloidal particles, from gelatin^22^, PLGA^31^, and dextran^32^, has been used to develop colloidal gels which are used as biomaterials for several applications. However, how the colloidal gels can be designed to control the matrix mechanomorphology and can be used to guide cellular morphogenesis via cell-matrix interactions has not been explored.

**Figure 1:**
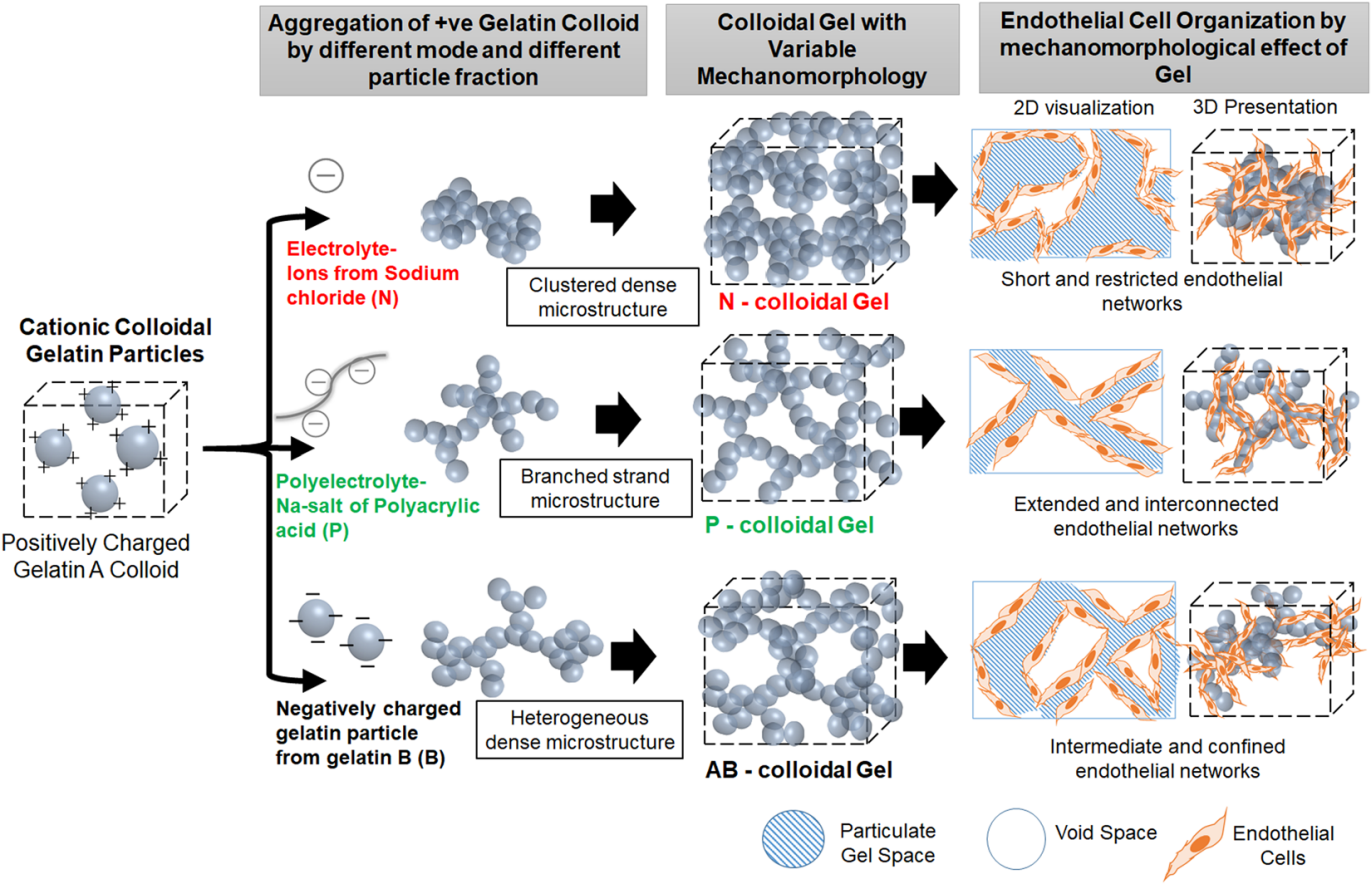
Mechanomorphological features of gelatin based colloidal gel to study endothelial morphogenesis. Different mechanisms of electrostatic interaction mediated aggregation of positively charged colloidal particle form different microstructure, with electrolyte mediated dense and polyelectrolyte mediated ramified network. Variance of microstructural morphology and gel stiffness combined can regulate endothelial cell organization.

In this study, we have developed gelatin-based colloidal gels from electrostatic interaction-mediated assembly of positively charged gelatin colloidal particles (from cationic gelatin A from porcine skin with isoelectric point ≈ 9) either through addition of electrolytes or negatively charged gelatin colloidal particles (from anionic gelatin B from bovine skin with isoelectric point ≈ 5), as described in Fig. 1. Neutralization of charge on the colloidal gelatin reduces the repulsive barrier and causes the particles to aggregate. By varying how the charges are neutralized, the aggregation mode of particles varies to yield different microstructural organization. Three approaches were used for the aggregation of cationic gelatin A colloid. In first approach, sodium chloride was used to aggregate the particles into compact clusters with constrained and less-interconnected void (referred as N-gel). In second approach, sodium salt of polyacrylic acid was used to induce simultaneous neutralization and bridging of the particles into tenuous strands with unconstrained and interconnected voids (referred as P-gel). Finally, oppositely charged gelatin B colloids were allowed to interact and aggregate into a heterogeneous dense mixed gel (referred as AB-gel). And as the particle fraction is increased in a given aggregation mode, the hierarchical evolution of the network in 3D leads to increased mechanical strength. Thus, gelatin based colloidal gel can present a range of 3D morphology which is controlled independent of matrix stiffness. Endothelial cells can be embedded within these gel matrices and the mechanomorphological cues from the matrix provide spatial guidance as well as mechanical signals to cells to regulate the vascular assembly. Moreover, gelatin based material ensured that these gel are cell-compatible and degradable, as it is derived from collagen and contains cell-recognizable functionalities e.g. RGD for cell adhesion and protease sensitive degradation sites for migration^33,34^.

Using this platform, we established the structure-property correlation of the colloidal gels and analyzed the response of ECs in gel matrices. Our results indicate EC networks are promoted in loose microstructured matrix formed by tenuous strands of particles due to increased focal adhesion and cadherin mediated cell-cell interactions, compared to dense microstructure. We further show that stiffness of the colloidal gels is modulated independently of matrix morphology and EC networks are reduced with stiffness of the matrix. Taken together, we have demonstrated that the colloidal gels can be used to regulate the mechanomorphology of matrices to influence EC morphogenesis, and potentially can be developed as therapeutic angiogenic material.

## Results and Discussion

### Aggregation of colloidal gelatin

**Figure 2:**
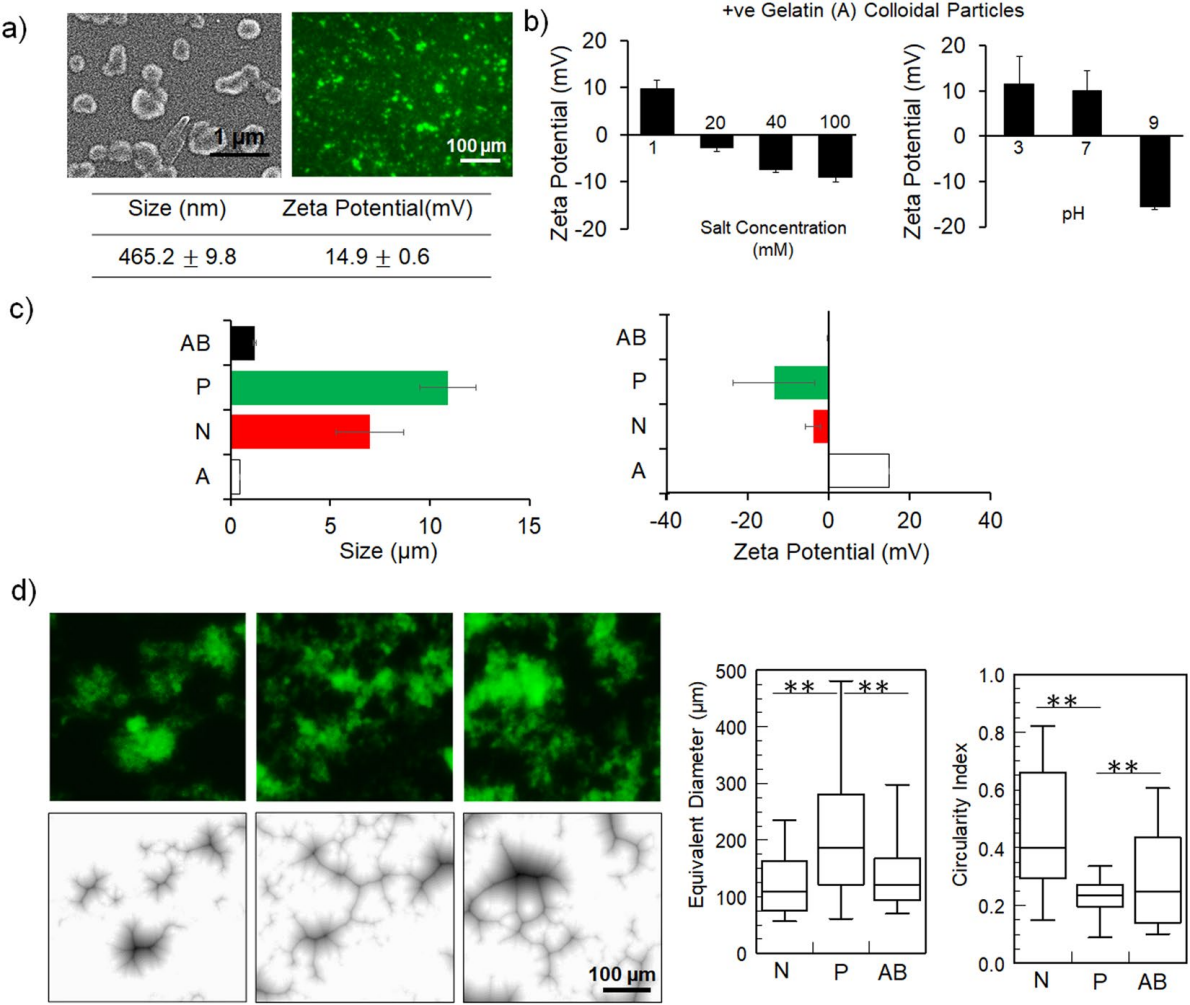
Electrostatic interaction mediated aggregation of colloidal gelatin particles. (**a**) Microscopic images of colloidal gelatin A particles from scanning electron microscopy and fluorescence images showing submicron particles which are uniformly dispersed. Average size and surface charge (zeta potential) measured by dynamic light scattering. (**b**) Variation of zeta potential of colloidal gelatin A particles as a function of ionic strength (from HEPES buffer at pH 7) and pH (with 1 mM HEPES buffer). (**c**) Size and zeta potential of aggregates following addition of 4 M sodium chloride (N), 10% (w/v) Na-salt of polyacrylic acid (P), and 1:1 negatively charged gelatin B (AB) particles showing aggregation of particles due to electrostatic interactions. (**d**) Morphological character of clustered network of N-, P- and AB- from fluorescence images and their corresponding distance map from ImageJ and their size (from equivalent diameter) and shape (from circularity index) analysis showing characteristic difference in particle organization in N-, P- and AB- aggregate (**P ≤ 0.01, ANOVA).

Positively charged colloidal gelatin particles were developed from cationic gelatin A using a desolvation method^35,36^ to form submicron particles (Fig. 2a), with an average size of 450 nm (and narrow size distribution with PDI below 0.2) and a positive zeta potential indicating positive surface charge. Similarly, the negatively charged colloidal gelatin B particles were submicron particles with an average size of 370 nm and negative surface charge (Fig. S1a). The fluorescent image also showed that the colloidal gelatin particles are stable and freely dispersed in aqueous media without any significant agglomerations due to the electrostatic repulsion. We further validated the presence of surface charges by measuring zeta-potential at different pH and ionic strength (by varying salt concentration). Figure 2b shows that positive zeta potential of gelatin A particles decreased (and ultimately reversed into negative potential) as pH and salt concentration increased. For gelatin B particles, increase in pH decreased the zeta potential (i.e. more negative) and increase in salt concentration increased the zeta potential (i.e. less negative), only at higher ionic strength (Fig. S1b). While both gelatin A and gelatin B particles shows screening of diffuse layer with increasing ionic strength, gelatin B particles are only screened at higher salt concentration due to the similar counterion of the added electrolyte. The effect of pH on the charge of colloidal gelatin particles are in accordance with the data reported^22,37^; whereas the effect of ionic strength on charge colloidal gelatin particles can be explained with respect to the data reported for organic and inorganic charged colloids^38–40^. Collectively, these data imply that colloidal gelatin particles are charges and stable due to their respective surface charges, and thus, represent the smallest unit for colloidal aggregation using electrostatic interactions.

To examine, if the electrostatic interaction mediated aggregation of gelatin A colloid can be induced by the addition of NaCl, PA and gelatin B particles (referred as N, P and AB respectively), we examined the change in size and surface charge following their addition using DLS (Fig. 2c), after the system equilibrated. For all three systems, aggregation was observed from the increase of size (which increased with time, Fig. S2a) and reduction of positive charge. Addition of NaCl at 4 M increased the size and lowered the charge indicating aggregation. In comparison, lower concentrations of NaCl (at 1 M and 2 M) showed relatively negligible change in size but reduced the surface charge (Fig. S2b). For PA induced interactions, the size increased and the surface charges reduced. These changes were proportional to the concentration of PA, showing maximum effectiveness at 10% of PA (Fig. S2b). Finally, when negatively charged gelatin B colloid was used to interact with gelatin A colloid, size increase and charge reduction occurred and the effective interaction occurred when the particle fractions were equal (i.e. 1:1) (Fig. S2b). Furthermore, size increase (and time-dependent change in size) was maximum in P and least in AB. The bridging interaction by PA increased the aggregate size due to its extended structure compared to N and AB, whereas AB showed minimum increase because it is formed by heteroaggregation of particles. Accordingly, N- and AB-type showed nearly complete charge reduction but P-aggregate showed negative charge from overcompensation due reentrant transition observed in polyelectrolyte mediated aggregations^41,42^. Thus, all the three approaches showed the electrostatic interaction mediated aggregation of colloidal gelatin A particles and determined the conditions for effective aggregation.

To analyze the microstructural organization of the colloidal particles into the aggregates by the three modes, we examined the fluorescent images of the aggregates after 2 hour of equilibration, using 0.05 particle fraction (w/v with respect to wet weight of gelatin particles). Compared to freely dispersed particles in the dispersion (Fig. 2a), all three approaches showed aggregation of the particles as seen in Fig. 2d. However, the pattern of organization was characteristically different in the three systems. N-aggregates were formed as compact dense clusters whereas P-aggregates were branched interconnected strands and AB-aggregates displayed a combination of both characters. This difference of structural organization, between three aggregate types, was more evident from the corresponding distal map images (generated from binary images using ImageJ) of the fluorescent images. Further analysis of the discrete aggregates showed that P-aggregates have significantly larger equivalent diameter but lower circularity (compared to N-aggregates and AB-aggregates), indicating that P-aggregates evolve as networks of tenuous strands due to the extended interconnections through bridging. Whereas, N-aggregates have smaller equivalent diameter and more circularity indicating compact dense sphere-like clusters and AB-aggregates are intermediate indicating both compact dense clusters and strand networks.

**Figure 3:**
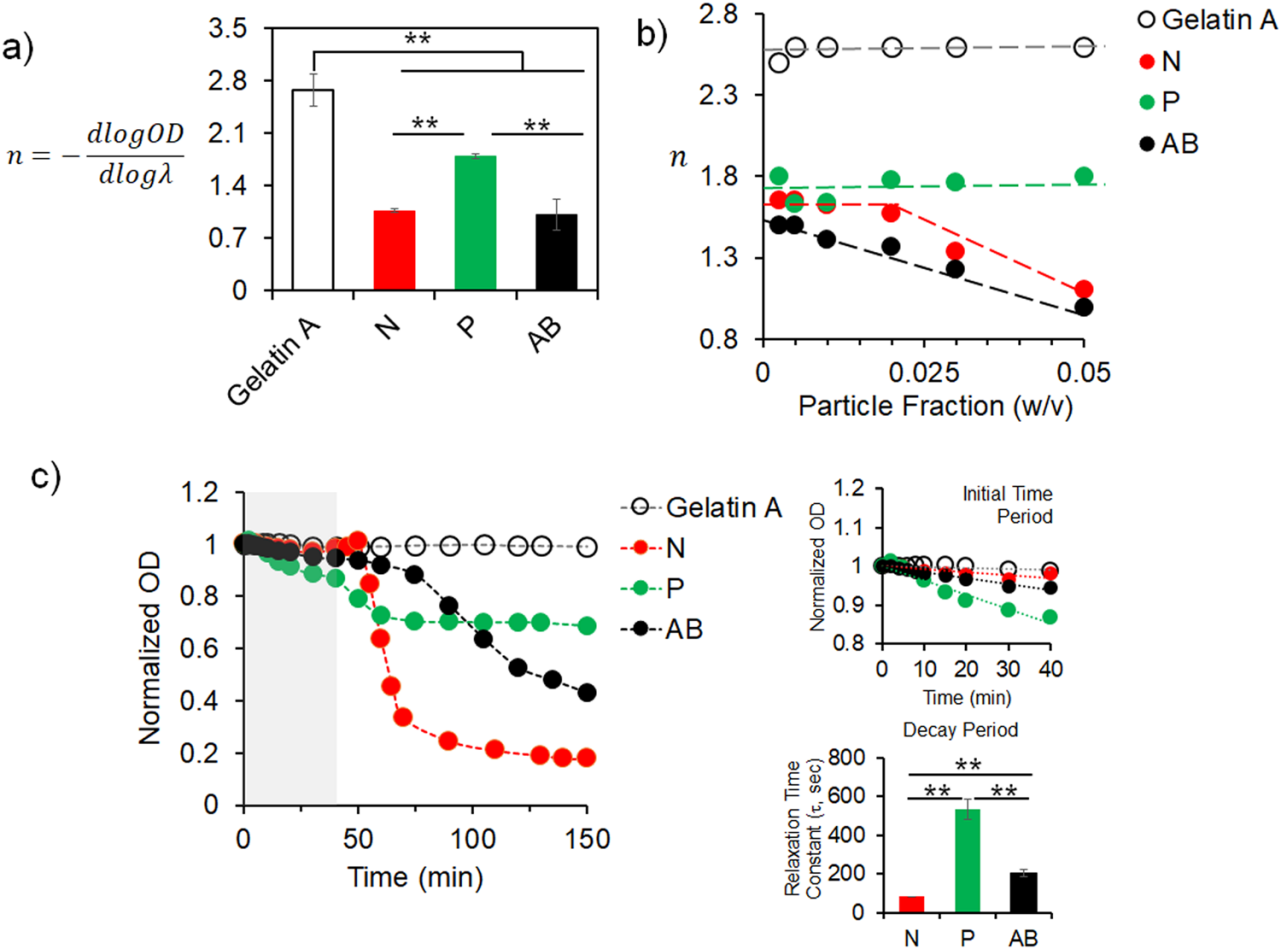
Aggregation mechanism and kinetics of colloidal gelatin particles. (**a**) Dispersibility factor ‘*n*’ of colloidal gelatin A particles and N-, P-, and AB- aggregates measured at 0.05 particle fraction, where decreased ‘*n*’ indicates aggregation (**P ≤ 0.01, ANOVA). (**b**) Variation of ‘*n*’ of colloidal gelatin A particles and N-, P-, and AB- aggregates with particle fraction shows difference in the dependence of particle fraction for aggregation (lines are estimate for guidance). (**c**) Kinetics of aggregation for N-, P-, and AB- aggregates (in comparison to non-aggregating colloidal gelatin A particles) measured at 0.05 particle fraction shows initial (shaded region) slow linear decrease followed by rapid decrease in optical density. Rapid decrease phase fitted with KWW function (see materials and methods) to extract the relaxation time constant for N-, P-, and AB- aggregates show highest relaxation time for P-aggregates (**P ≤ 0.01, ANOVA).

To further validate the aggregation and their modes in these systems, we performed turbidimetric measurements to calculate the ‘dispersibility factor, *n*’ based on the wavelength dependence of the turbidity of the particles, from the slope of log[turbidity(OD)] versus log[wavelength(*λ*)] curve (Fig. S3); where *n* value decreases with particle aggregation and growth of aggregate size, because aggregation and growth of clusters lowers the turbidity and its dependence on wavelength^43,44^. At 0.05 fraction, *n* decreased significantly for all three aggregates compared to gelatin A colloidal dispersion, indicating aggregation (Fig. 3a). But *n* value of P-aggregates were higher than that of N- and AB- aggregates indicating that the turbidity of P-aggregates was not lowered to the same extent as it occurred for N- and AB- aggregates. This is due to the strand-like microstructure of P-aggregates which are mainly extended compared to more compact N- and AB- aggregates. This was further corroborated from the change of *n* with respect to particle fraction (Fig. 3b). Colloidal gelatin A dispersion showed constant *n*; compared to that P-aggregates showed lower but no change in *n* whereas N-aggregates showed a change at certain particle fraction and AB-aggregates showed decreasing *n* at a low particle fraction. P-aggregates attain relatively smaller characteristic dimensionality with increasing fraction because P-aggregates are extended as strands, due to which *n* value remained unchanged over the measurable range of particle fraction. N aggregates showed a change in *n* at a critical particle fraction because at low fraction although NaCl reduces the repulsive barrier but the particle fraction is not high enough to show significant growth of aggregates, and beyond the critical fraction aggregates grow in multiple dimensions as compact cluster. Interestingly, AB aggregates demonstrated most pronounced effect because the aggregates are initiated by particle-particle interaction. Both *n* and change of *n* w.r.t. particle fraction showed that electrostatic interaction mediated assembly of colloidal gelatin A by different modes forms different microstructured clusters. Finally, the kinetics of aggregation was measured from OD vs. time graph (Fig. 3c). As expected, there was no change in OD of pure colloidal gelatin A because the colloidal particles are stabilized in dispersion due to electrostatic repulsion. For all the three modes, change of OD owing to aggregation followed two distinct patterns: the initial period (shaded region in Fig. 3c) followed by relatively rapid decrease of OD. The initial period showed slow decrease of OD which was less than 20% of its initial value and the following period showed rapid decrease of OD owing to relaxation of structures as the aggregates evolved. During the initial period, P-aggregates showed faster decrease in OD as the polyelectrolyte through multiple sites bridged the particles to aggregate, typical of fast diffusion limited aggregation, whereas, N- and AB- showed slower decrease as the particles require time to overcome the energy barrier for aggregation, typical of slow reaction limited aggregation^1,30,45^. This data corroborates the DLS based analysis of size which showed rapid increase of P-aggregate size (Fig. S2a). Following the initial period, OD decreased rapidly in N- but slowly in P- and AB- shows an intermediate rate. Average time for relaxation (τ) was obtained from the fitting of the kinetic data into Kohlrausch-William-Watts (KWW) function for the decay period: $y(t)={e}^{{(-\frac{t}{\tau })}^{\beta }}$, where *y*(*t*) is the normalized OD with respect to the OD at *t* = 0, and *τ* is mean relaxation time and *β* is relaxation distribution parameter. KWW function is used to analyze polymer, colloids and protein aggregation and provides a tool to measure the aggregation from the kinetics of the transformation from a non-equilibrium to an equilibrium state^46^. P-aggregates exhibited four times longer relaxation time compared to N- and AB- aggregates. Following initial cluster formation, the bridging interactions in P-aggregates require longer time to structurally evolve as strands whereas N- and AB- can grow at a shorter time-scale. The two step change in turbidity indicates N-aggregates evolves slowly in a reaction-limited mode as compact aggregates which ultimately relaxes rapidly. In contrast, P-aggregates are formed rapidly in a diffusion-limited mode as ramified strands which requires longer time to relax. AB-aggregates follow a combined approach leading to heterogeneous structure. As a result, overall turbidity decrease was highest in N- but the relaxation time was highest for P- aggregates. Similar responses were observed from the aggregation of cationic starch particles with different chain length of waxes^46^. This kinetics data further support the mechanism of aggregation induced by the three modes to develop different microstructured colloidal clusters.

The combined evidences from DLS, microscopic images and turbidimetric analysis demonstrate that electrostatic interaction mediated aggregation of colloidal gelatin A leads to cohesive aggregates of different morphological patterns according to the mode of aggregation. Mechanistically, perturbation of diffused layer of ionic colloids with increasing ionic strength or charge neutralization reduces the repulsive potential leading to aggregation. The particle-particle contact in N- is followed by the screening of the diffused layer and therefore, the aggregation is reaction limited. Whereas in P-, polymer chain due to its connectivity simultaneously bridges (i.e. interconnects) and reduces the surface charge, and the aggregation is diffusion limited. Both of these modes of colloidal aggregation has been observed in experimental and simulated models^47–50^, which supports the colloidal aggregates of gelatin system. However, there can be a crossover between these two modes dependent on stage of aggregation (as well as on the concentration of electrolyte and particle fractions, and their combined effect). Overall, microstructural morphology of N-aggregates is compact dense while P-aggregates are branched strands and AB-aggregates show heterogeneous anisotropic structure. It is also important to note that we have not varied any physical conditions (e.g. aggregation time, shear force, temperature) to modulate the gelation because it is known that physical conditions can modulate gel structure^51–54^. Therefore, these gelatin based aggregates are morphologically distinct owing to their mode of aggregation and provide a platform to use these materials as macroscopic gel where both morphology and mechanics can be modulated.

### Mechanomorphology of colloidal gelatin gel

**Figure 4:**
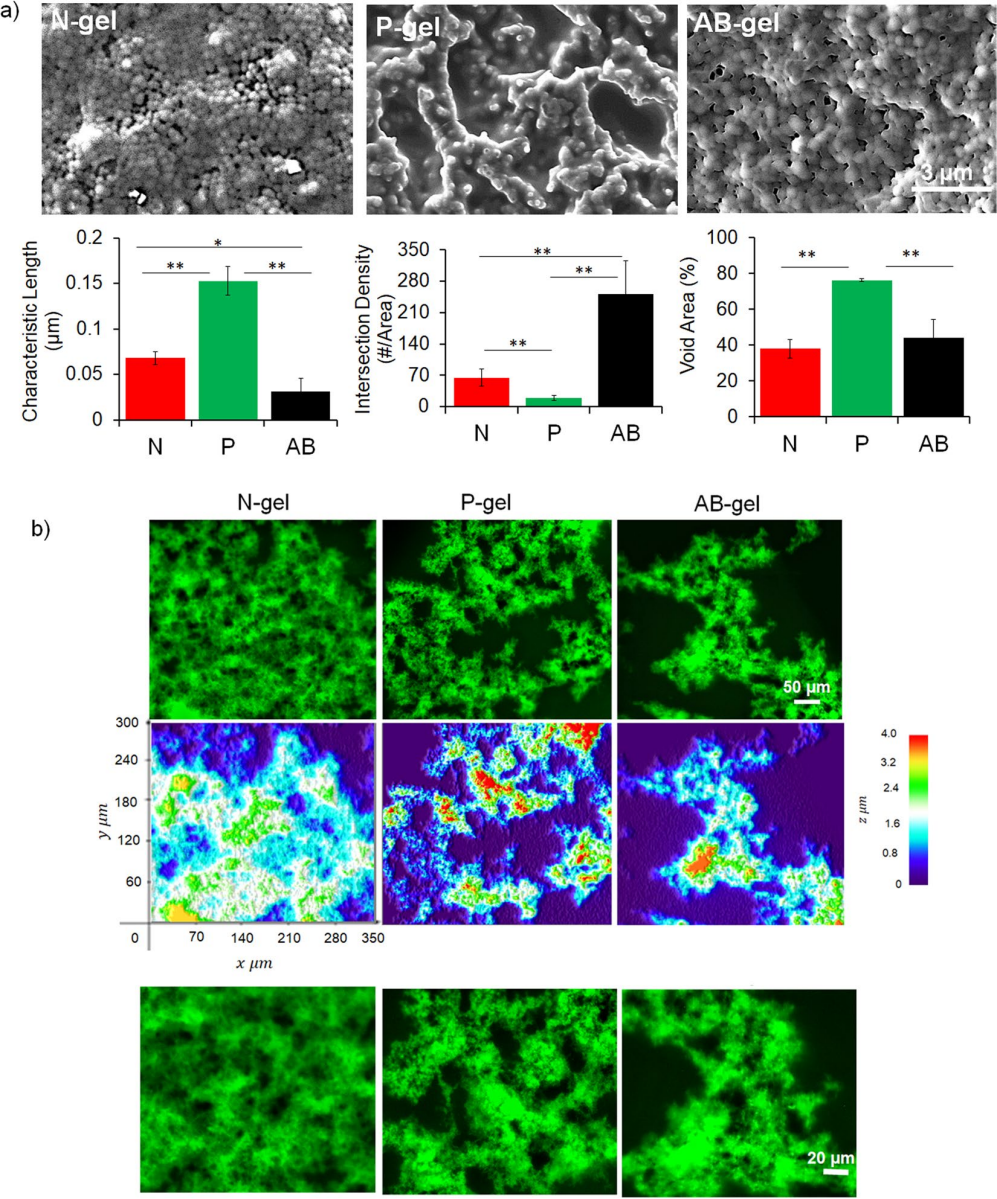
Morphology of gelatin based colloidal gels. (**a**) Microstructured morphology of N-, P-and AB-gel from scanning electron microscopic images. Morphological features of the microstructure analyzed by measuring characteristic length, intersection density and void areas of the gels from these images using DiameterJ plugin in ImageJ (**P  ≤ 0.01, *P  ≤ 0.05, ANOVA). (**b**) Confocal scanning fluorescent images and 3D interactive surface plot (color code indicates z-depth) of gels showing morphology and spatial distribution of voids in the gels. Lower panel images are enlarged version for better visualization.

To use these aggregated clusters as colloidal gel, we developed the gels with three different particle fractions for all the three types of aggregates. For a given mode, as the particle fraction is increased, the aggregate of interconnected particles grows in 3D and the elasticity increase, because elasticity of the particle networks (i.e. clustered particles) is proportionate to the length and composition^55–58^. Although the elasticity increase, morphologically colloidal gels are self-similar in 3D because the microstructural organization of the colloidal particles evolve in a similar pattern as the network grows. We varied the particle fraction below the percolation threshold (the condition at which the colloidal network span to occupy the entire volume) and used the aggregated clusters at three different fractions as gels for N-, P- and, AB- aggregates. Morphological characterizations of the gels were performed with SEM and confocal imaging for all three gels developed from 0.05 particle fraction. SEM images (Fig. 4a), which provided microscale organization of the particles in the gel, demonstrated the aggregation of particles with distinctly different morphologies for the three gels. N-gels displayed compact aggregation with relative less voids whereas in P-gels the particles were organized as strands with more void areas. In comparison, AB-gels showed compact dense structure which resembled more like N-gels and displayed some local voids. This observation corroborated the structural analysis of individual aggregates (in Fig. 2d), providing the evidence that the structural organization of colloidal clusters is evolved into the gels. We further analyzed the SEM images using ImageJ (with DiamterJ plugin) to extract the structural parameters by measuring characteristic length of particle networks (in between the intersections), intersection density and void area^59^. The network of interconnected particles was significantly longer in P-gel because the particles were aggregated as strands, whereas in N- and AB-gel the particles were organized in compact form resulting in a shorter length. Compared to N-gel, network lengths were shorter in AB-gel due to the heterogeneous random particle aggregation. Strand like elongated networks of P-gel formed fewer intersections (in between two or more networks) compared to N- and AB-gels; whereas AB-gels displayed maximum intersections due to its random structure. Finally, due this morphological organization the void areas in P-gels were highest compared to other ones.

While SEM images provided information at scale length relevant to individual cell, we performed confocal imaging of the gels to capture the morphological features at a larger length scale (hundreds of microns) relevant to multicellular dimension. Confocal images (with a zoomed view in lower panel images) and their corresponding 3D surface interactive plot (generated from z-stacks using ImageJ) in Fig. 4b show that the macroscale morphology is translated from the organization of particles in the respective gels. N-gels were dense with less and constricted voids whereas P-gels were evolved with interconnected strands with more extended and connected voids, and AB-gels showed characteristics heterogeneous features where some regions are dense and some are dispersed. This variation of morphology in these gels essentially creates a significantly different spatial microenvironment (at multicellular dimension for cells to organize) owing to the organization of the particles. These features are self-similar with increasing particle content because the network expands in 3D in a hierarchical manner, due to which the similar morphological features were observed at the same length scale, when the gels were formed at 0.2 particle fraction (Fig. S4).

**Figure 5:**
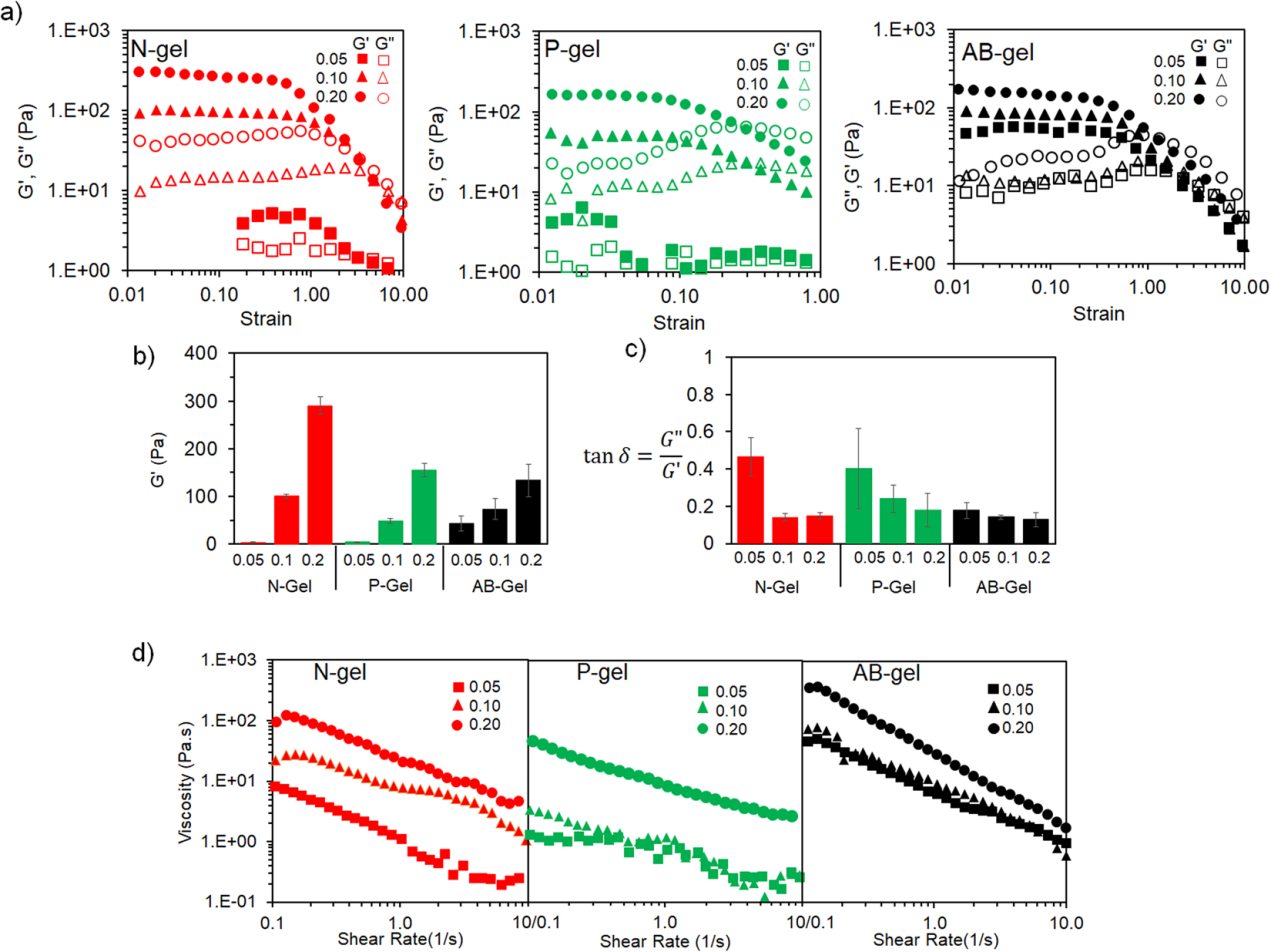
Mechanical characterizations of colloidal gel from rheology. (**a**) Strain amplitude sweep of N-, P-and AB-gel at different particle fractions measured at constant frequency. (**b**) Variation of elastic moduli (G′) and (**c**) tan(δ) of N-, P- and AB-gel with different particle fractions measured from linear viscoelastic region. (**d**) Viscosity and shear thinning behavior with respect to shear rate of N-, P- and AB-gel with different particle fractions.

To analyze the mechanical properties, we studied the rheological responses and correlated to the colloidal gel structure. Oscillatory strain sweep at constant frequency revealed that all the gels are displaying solid like response with storage (elastic) modulus (G′) greater than loss (viscous) modulus (G″). Typical strain amplitude response of N-, P- and AB-gels formed with different particle fractions are shown in Fig. 5a. With increase in particle fraction, all gels showed an increase in G′ (Fig. 5b) as network structure extended with the particle aggregation. Elastic moduli of the N- and P-gels at 0.05 fraction were around 5 Pa, while AB-gel showed 40 Pa. As particle fraction increased, elastic modulus of N-gel increased to 300 Pa (i.e. 60 times increase) whereas that of P-gel increased to 150 Pa (i.e. 30 times increase). Scaling of elastic moduli with particle fraction was most pronounced for N-gel because the networks evolved from the compact dense organization of particles, whereas for P-gel the networks grew as strands of interconnected particles. This can also be explained in terms of longer characteristic length and fewer interconnections of networks in P-gels compared to that of N-gel. In contrast, AB-gel showed least dependence on particle fraction indicating heterogeneous anisotropic growth of networks. This interrelationship between particle fraction and moduli indicates a self-similar growth of the networks in 3D for colloidal gels^57,58^, in contrast to uncontrolled microstructure of mesh-like hydrogels. N- and P-gels evolved in homogeneous manner to maintain similar microstructure across several length scales, which can create a spatially uniform microenvironment for cells. In contrast, the heterogeneous microstructure of AB-gel, which is inherently devoid of self-similarity, showed less scaling of moduli with particle fraction. These characteristics are also evident from the analysis of tan(δ) as shown in Fig. 5c. All gels at all particle fractions showed a value lower than one indicating elastic moduli higher than viscous moduli. At 0.05 fraction, N- and P-gel showed higher tan(δ) because small size clusters enables movement of interstitial fluid within the cluster (which are homogeneous) while similar behavior was prevented by heterogeneity of microstructure in AB-gel. As particle fraction increased, tan(δ) decreased for all N- and P-gels due to the growth of network indicating dominance of elastic solid phase, while tan(δ) of AB-gel practically remained unchanged due to the randomness in microstructure. Compared to the reported gelatin based colloidal gel^22,60^, elastic moduli of the gels in this studies were lower because the particle fractions used here were based on swelled particle rather than the absolute dry weight basis. Additionally, the viscoelastic moduli (i.e. G′ and G″) were measured in presence of cell culture media and in physiological temperature for all three gels, at 0.05 and 0.2 fractions (since these gels were used for endothelial morphogenesis) to ensure if there are any variations due to change in the conditions. Results show (Fig. S5) that the elasticity and viscoelastic responses of the colloidal gels were not altered under this condition.

Further insight into the mechanomorphology was assessed by analyzing the viscosity of the gels with respect to the shear rate (Fig. 5d) and by comparing their power-law index ‘*n*’ and consistency ‘*K*’^61^ (Table S1). All the gels showed a shear thinning response with increased shear rate, however the responses were dependent on the gel type. P-gels showed enhanced flow behavior because the strand-like networks can easily deform and the interstitial fluid can flow through the interconnected voids with increasing shear rate, compared to compact dense N- and AB-gel. Furthermore, as the particle fraction was increased for a given gel, flow behavior decreased because the extended networks can resist deformation. Particularly, at low particle fractions, P-gels reached a Newtonian plateau as the shear rate increased, indicating that the smaller strands of P-gel were deformed to respond as fluid, whereas N- and AB-gels showed continued shear thinning. Higher ‘*n*’ and lower ‘*K*’ of P-gels compared to N-and AB-gel further supported this analysis. Thus, the shear rheological measurements of the three gels provided supportive evidence for the mechanomorphology of the gels.

**Figure 6:**
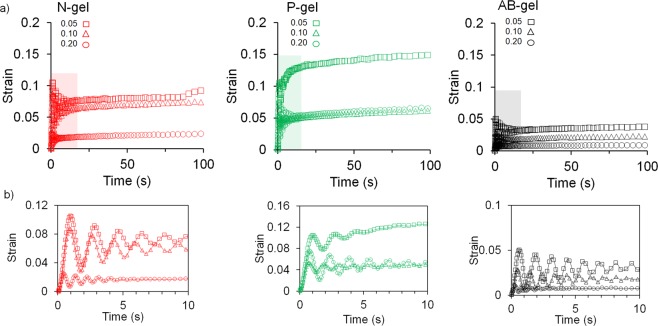
Time-dependent viscoelastic response from creep measurement. (**a**) Deformation of N-, P- and AB-gel with different particle fractions at constant stress of 2 Pa for 100 s. (**b**) Creep ringing phenomena from damped oscillation in strain observed at early creep time within 10 s of the gels.

Finally, we measured the creep response at constant stress (below the yield) by measuring the deformation of the gels to analyze the time-dependent changes. Creep response of colloidal gels depends on the mechanics and the morphology^55,62–64^, because the network structures of colloidal gels develop from the aggregation of particles. P-gels due to their strand-like elongated networks experienced significant deformation (Fig. 6a), almost 15% after 100 s with 0.05 particle fraction, as the inter-particle bonds within strands of P-gels are relatively easily extended and dislocated (under stress), and thus enabling the flow. As particle fraction increases, the creep responses are decreased because the larger networks prevented the deformation^55^. N-gel showed relatively lesser creep response under similar conditions due to its compact microstructure with less than 10% deformation. Moreover, creep of N-gels almost plateaued after the instantaneous strain whereas P-gel continued to deform with time. In comparison, AB-gel showed least deformation due to its heterogeneous microstructure which is relatively random and anisotropic in 3D. Figure 6b shows the creep response of the gels during the initial time (t < 10 s), where creep ringing responses were observed. Creep ringing phenomena arises due to inertial effect but is also reflective of gel mechanomorphology^65,66^. Ringing response is more pronounced in dense gels with closed structure and is also dependent on interfacial effect from the movement of interstitial fluid^67,68^. P-gel due to its open structure showed lesser oscillation compared to N- and AB-gel which are denser. As, the particle fraction increased P-gels showed increased ringing as networks are evolved and due to the greater ability of interstitial fluid to flow through interconnected voids. Whereas, for N- and AB- gels, at 0.2 particle fraction, ringing responses were lower (compared to 0.05 particle fraction) which can be attributed to the less mobility of fluid.

Overall, this data collectively shows that gelatin based colloidal gels can be engineered through electrostatic interaction mediated aggregation of particles. Depending on the mode of aggregation and the particle fraction, these gel show a tunable combination of microstructural morphology and mechanical properties. Morphologically, N-gels are compact and dense while P-gels are branched strands network, and AB-gels show heterogeneous anisotropic character. As these gels evolve with particle aggregation, these microstructures are self-similar and is independent of the particle fraction from which the gels are formed. But as the particle fraction increased, stiffness of the gels increases due to the growth of the network structure. Elastic moduli of N-gels showed increased scaling with particles due to its compact microstructure compared but P-gels showed relatively lesser enhancement of moduli; and AB-gel due to its heterogeneity showed least dependence of moduli on particle fraction. Thus, gelatin based colloidal gels provide a uniquely complete system to tune the mechanomorphological properties in a controlled and independent manner.

### Endothelial morphogenesis in colloidal gel

**Figure 7:**
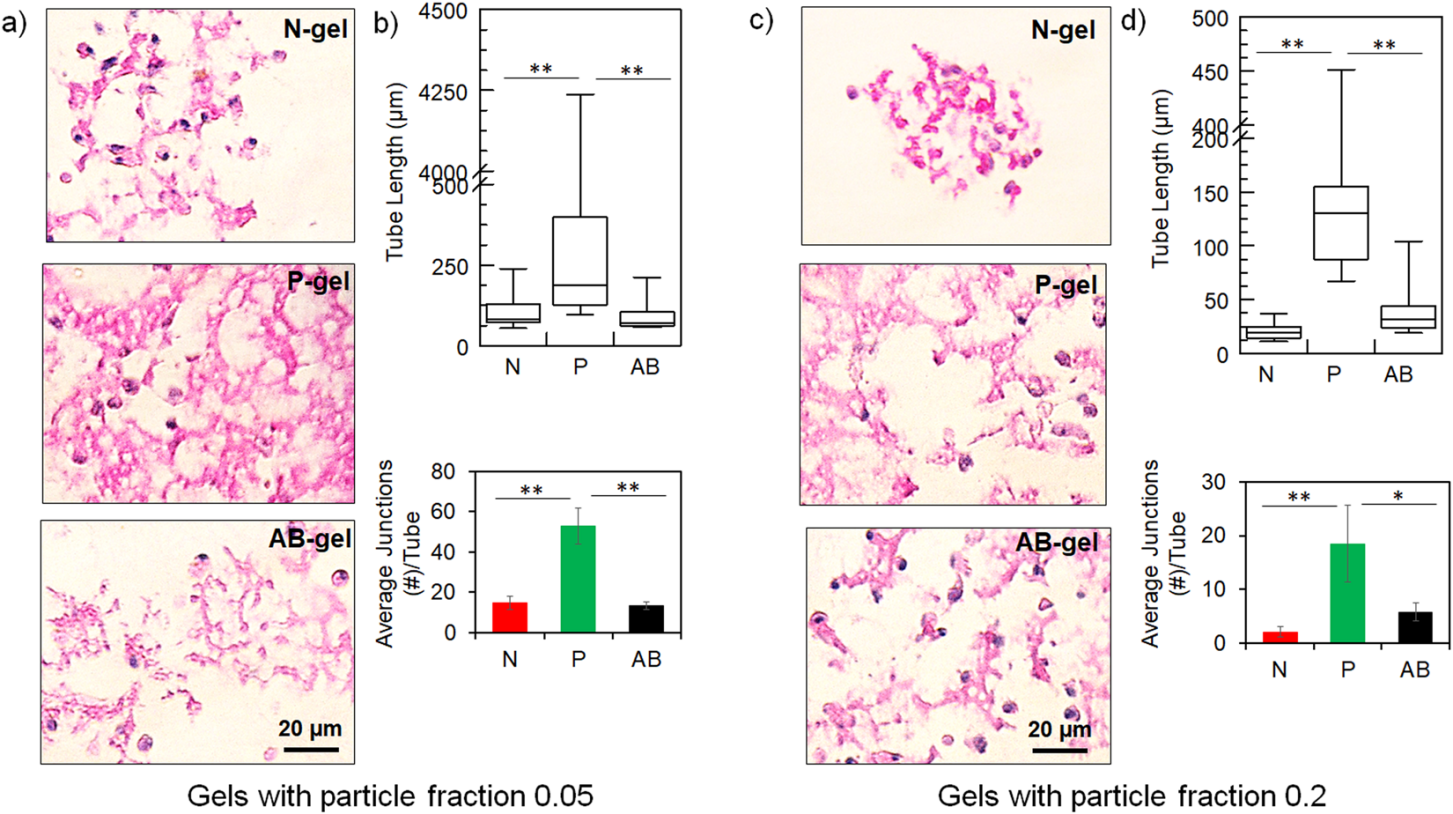
Endothelial morphogenesis in colloidal gel. Morphogenesis of endothelial cells in N-, P- and AB-gel (**a**) with 0.05 particle fraction, and (**c**) with 0.2 particle fraction, at 48 hrs from H&E stained images. Quantification of endothelial network structure from tube length and numbers junction per tube in N-, P- and AB-gel (**b**) with 0.05 particle fraction, and (**d**) with 0.2 particle fraction, at 48 hrs analyzed from H&E stained images after skeletonization of the images using ImageJ (**P ≤ 0.01, *P ≤ 0.05, ANOVA).

To analyze the mechanomorphological effect of colloidal gels on endothelial organization, we embedded HUVEC in N-, P-, and AB-gels developed from low (0.05) and high (0.2) particle fractions. In all conditions, cells exhibited similar viability (measured by quantifying DNA content, Fig. S6), inducing no apparent adverse effect on cell responses. Organization of ECs were assessed from histologically stained images, and the quantified parameters of EC network showed significant differences. At low particle fraction, ECs in P-gels showed extensive interconnected endothelial tube-like network structures. By comparison, cells in N- and AB-gel networks were shorter and disconnected (Fig. 7a). Accordingly, tube lengths and the average number of junctions per tube (indicating the extent of branching in EC tubes) were significantly higher in P-gels compared to N- and AB-gel (Fig. 7b). Specifically, in P-gels, the connectivity of ECs in extended beyond 1 mm, indicating extended interconnections between the cells. At high particle fractions, similar trends were observed between the three gels, but extent of network formation decreased considerably in all the gels compared to their low particle fraction counterparts. Figure 7c shows ECs in high particle fraction gels, where ECs in N-gels showed less connectivity and remained dispersed. By comparison, cells in P- and AB-gels showed more networks, with cells in P-gels showing more extended networks than AB-gels. The length of EC tubes and the average number of junctions per tube (Fig. 7d) were highest in P-gels compared to others. However, compared to the low particle fraction gels (Fig. 7b), both of these features of the EC network decreased by 2- to 3-fold in their respective gels.

At low particle fractions, N- and P-gels exhibited similar elasticity (which is low ~5 Pa) but EC networks were highly elongated and branched in P-gels, indicating that matrix microstructure guided ECs to form networks. The strand-like ramified microstructure and unconstrained voids of P-gels provided directional guidance and spatial microenvironments for cells to organize and interconnect into extensive networks, whereas the dense matrix and localized voids of N-gels restricted the ability of cells to connect. In AB-gel, EC networks were similar to that of N-gels, indicating that heterogeneous and relatively dense microstructures of the AB-gels inhibited EC networks, although this can also be due to relatively higher stiffness (40 Pa compared to 5 Pa of N- and P-gel).

As particle fraction increased, the elastic moduli of all three gels increased but their respective microstructural morphology remained similar (compared to their corresponding low particle fraction i.e. soft gels). In this case, ECs showed reduced network formation. This is expected because increased matrix stiffness reduces EC organization into tubular networks, as reported in different types of gels^69–71^. However, the effect of increased stiffness in these reported studies is due to the increased matrix density whereas in colloidal gels it is due to the increased network size of particle aggregates without a change in the matrix morphology. This phenomenon becomes evident when EC organizations were analyzed in high moduli gels developed from high particle fraction. Among the high particle fraction (i.e. high modulus) matrices, P-gels showed the most extensive EC networks compared to N- and AB-gel. In N-gels, a decrease in EC network was observed with cells remaining localized and without connection, which is collectively attributed to dense microstructure and high stiffness (~300 Pa). However, P- and AB-gel exhibit similar elasticity (~140 Pa) but more extensive EC networks in P-gel indicates that branched strands and interconnected voids of P-gel facilitated EC organization into tube-like networks compared to the heterogeneous (anisotropic) dense matrix of AB-gels. Thus, tenuous strands and spacious voids of 3D matrix is the most permissive microstructure to promote EC network irrespective of matrix stiffness. The effect of microstructure mediated EC morphogenesis can be further explained in terms of time-dependent viscoelastic response^72^. P-gels exhibit noticeable time dependent changes due to its tenuous strands and spacious voids at all particle fractions (compared to corresponding N- and AB-gels) and can promote EC networks.

Collectively our results support the fact that dense and mesh-like matrices prevent cell extension and migration^19,21,73^. Moreover, cells including ECs, require tracks either through aligned matrix and void space to migrate and form functional structures like tubes and lumen^74–76^. Our results show that ECs can form networks in stiffer matrices if the matrix is microstructure is loose but are likely inhibited from forming network in a soft matrix if the matrix is dense in microstructure. In fact, comparison of EC networks in low fraction N-gel and high fraction P-gel showed similar extent of EC organizations (Fig. S7). This demonstrated a bidirectional counteractive correlation between matrix microstructure and morphology, where the supportive microstructure can attenuate the inhibitory effect of matrix stiffness during endothelial morphogenesis.

### Cell-matrix and cell-cell interactions in endothelial morphogenesis

**Figure 8:**
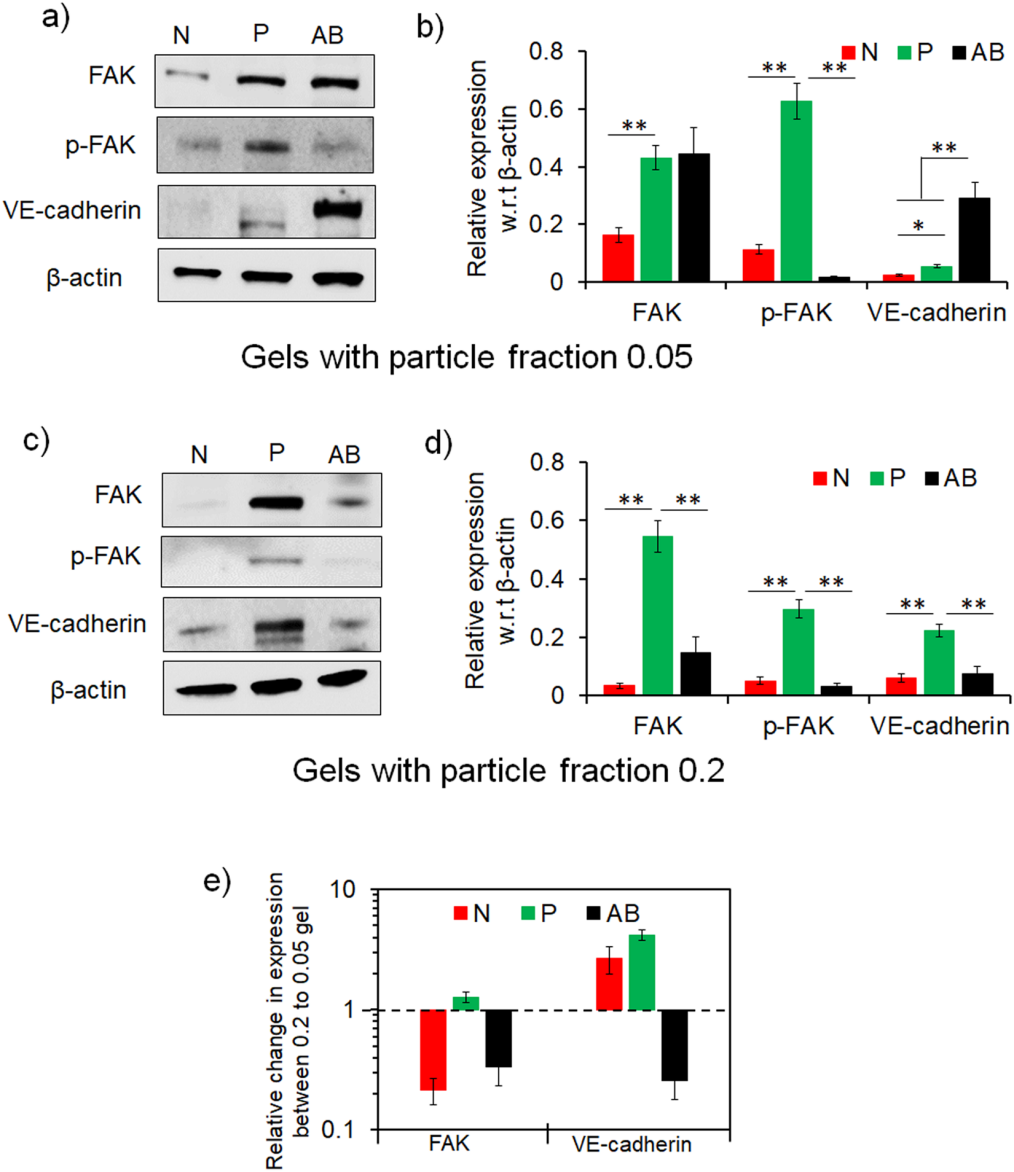
Cell-cell and cell-matrix interactions guide endothelial morphogenesis in colloidal gels. (**a**) Levels of focal adhesion kinase (FAK), phosphorylated FAK (p-FAK), and VE-cadherin in endothelial cells measured by immunoblot analysis. β-actin as loading control in N-, P- and AB-gel (**a**) with 0.05 particle fraction, and (**c**) with 0.2 particle fraction, at 48 hrs. Quantified level FAK, p-FAK and VE-cadherin normalized to β-actin shows differential level of markers in endothelial cells in N-, P- and AB-gel (**b**) with 0.05 particle fraction, and (**d**) with 0.2 particle fraction, at 48 hrs (**P ≤ 0.01, *P ≤ 0.05, ANOVA). (**e**) Relative change of FAK, and VE-cadherin in endothelial cells in N-, P- and AB-gel with 0.2 particle fraction with respect to gels with 0.05 particle fraction. The corresponding full length blots are represented in Supplementary Fig. S8.

Differential EC responses in colloidal gel matrices due to the mechanomorphology indicate that the cell-matrix and cell-cell interactions were modulated during capillary-like network formation. To examine these interactions, we analyzed the expression of focal adhesion kinase (FAK), its phosphorylation at Tyr^397^ (p-FAK) as a representative marker for cell-matrix adhesive interactions and VE-cadherin as a representative marker for cell-cell interactions. FAK (and p-FAK) are signature expressions due to integrin mediated focal adhesion to matrix and cadherin mediated homotypic adhesion between cells^77–80^. Figure 8a shows representative western blots and Fig. 8b shows expression levels of the markers in the gels at 0.05 particle fraction while Fig. 8c shows representative western blots and Fig. 8d shows expression levels of the markers in the gels at 0.2 particle fraction.

At low particle fraction, FAK was expressed at a significantly higher level and was significantly activated from phosphorylation at Tyr^397^ in P-gels compared to N-gels. Since both gels exhibit similar elasticity, the difference in FAK expression may be attributed to the gel morphology. The ramified fiber-like strands of P-gel provide more accessible surface to cells, which enhances matrix adhesion, leading to increased FAK phosphorylation^79,81^. This facilitates cell elongation and motility in P-gels to form extended tubular EC networks, whereas clustered dense microstructure prevented similar responses in N-gels. VE-cadherin expression in P-gel was higher than N-gel suggesting that the extended EC tubes were stabilized through cell-cell adhesion^82^. Both cell-matrix and cell-cell adhesions were balanced from upregulation of FAK and VE-cadherin respectively, to mediate EC capillary networks in P-gels. Interestingly, AB-gel showed FAK expression at a similar level compared to P-gels but without sufficient FAK phosphorylation. This indicates that the focal adhesions in AB-gels were not active and mature enough to extend EC networks. However, VE-cadherin expression was highest in AB-gels, which apparently is contradicting but likely indicates that heterogeneous anisotropic morphology induced localized cell-cell adhesion.

At higher particle fraction, P- and AB-gels exhibited similar elasticity (~140 Pa), whereas N-gel was stiffer (~300 Pa) than both. FAK and p-FAK expressions were significantly higher in P-gel compared to N- and AB- gels indicating that the microstructure of P-gel favored cell-matrix interactions to promote EC networks. Since the elasticity of AB-gel is comparable to that of P-gel but their microstructures are distinctly different, upregulation of FAK and p-FAK in P-gels showed that EC networks were favored through increased adhesion on the tenuous strands of gel networks. Interestingly, VE-cadherin expression was also higher in P-gels compared to AB-gels which shows EC tubes in P-gels are also stabilized from cell-cell adhesion. In comparison, compact N-gel with increased stiffness showed decreased FAK expression (and without detectable phosphorylation) which significantly reduced cell-matrix interactions; as cells were highly constrained but likely induced some localized cell-cell adhesions (through VE cadherin expression) to form discrete short EC tubes. Since the microstructural morphology of colloidal gel controls the time-dependent viscoelastic responses, the differential expression of these markers can be correlated to the time-dependent changes. P-gels owing to its microstructure exhibits noticeable time-dependent changes which can enable the cells to extend and increasingly interact with matrix^72^. This feature can increase FAK activity to promote adhesion and form EC networks.

Finally, the relative changes in FAK and VE-cadherin expressions in stiffer N-, P- and AB-gels (i.e. gels at 0.2 particle fraction) with respect to their softer counterparts (i.e. gels at 0.05 particle fraction) are analyzed in Fig. 8e. For N-gel, FAK expression was reduced and VE-cadherin was increased since dense microstructured matrix with high elasticity inhibited effective matrix adhesion by restricting cell-matrix interactions. This in turn can induce cadherin mediated cell-cell interaction. In P-gels, total FAK expression remained unchanged, but activation of FAK through phosphorylation was somewhat reduced, indicating lesser effective matrix adhesion. However, VE-cadherin was upregulated indicating cell-cell adherence were more matured. In contrast, both FAK and VE-cadherin were downregulated in AB-gel. While reduction of FAK was similar to N- and P-gels indicating inhibition of matrix adhesion, but lower VE-cadherin was somewhat unexpected. Since heterogeneity and anisotropy of microstructure in high particle fraction AB-gel is amplified due to growth of particle networks, it can restrict cell-cell interactions to downregulate VE-cadherin. In general, as the elasticity of the colloidal gels increased, FAK or p-FAK activity was reduced indicating lesser extent of cell-matrix interactions. This effect corroborated the reduced EC networks in all the gels (Fig. 7c,d). In 2D studies, it has been shown that FAK and activated FAK is upregulated with substrate stiffness^83,84^, but in 3D systems focal adhesion activity have shown to be context dependent with variable responses^85^. In colloidal gels, increase of stiffness owing to increased particle fraction does not alter the microstructural morphology, but reduces the time-dependent changes due to larger particle clusters. It is likely that this feature counteracts the effect of increased stiffness to promote FAK activity in colloidal gel, as opposed to other systems. Interestingly, both FAK and VE cadherin expressions were higher in loose but stiffer (i.e. P-gel from high fraction) matrix compared to dense but soft (i.e. N-gel from low fraction). This implied that loose spacious matrix favors functional EC network by attenuating the effect of stiffness through a balanced cell-matrix and cell-cell adhesion, even though the EC networks were morphometrically similar.

Overall, at a given elasticity, either soft or stiff, cell-matrix interactions are favored with enhanced FAK activity when the matrix microstructure from the aggregated particles form ramified strands to provide increased matrix adhesion and cell-cell adhesions are stabilized from concomitant VE-cadherin expression. This shows cell-matrix and cell-adhesion acted in parallel to favor EC networks. Whereas, for a given microstructural morphology, as particle fraction increased the gel stiffness, FAK and/or FAK activation was reduced indicating lesser adhesion to matrix but it enhanced VE-cadherin, implying an antagonistic relation between cell-matrix and cell-cell adhesion. This bidirectional relationship indicates a difference in mechanism of focal adhesion-cadherin crosstalk in ECs due to the combined effect of matrix morphology and stiffness.

This comprehensive analysis of endothelial morphogenesis with gelatin based N-, P- and AB-gel decoupled the effect of 3D matrix mechanics from its morphology to reveal their independent roles on EC network formation and to underline the relevance of cell-matrix and cell-cell interactions. Differential expressions of FAK (and p-FAK) and VE-cadherin show that ECs can sense the matrix mechanomorphology to regulate these interactions in a distinct manner during network formation. However, these interactions are interrelated in a complex way and are linked to multiple downstream signaling pathways, associated with different markers, e.g. Rho family of GTPases (Rho, Rac and Cdc42)^77^. Complete analysis of the associated markers and pathways is necessary to understand how ECs respond to the colloidal gel matrices. It is also important to mention that these morphogenetic features and expression of markers are early time responses and long-term responses are essential to completely understand this effect in terms of network maturation and matrix remodeling. Also, this study is based on HUVEC, it is important to verify these responses with different endothelial cells to generalize these responses.

## Conclusions

In summary, we have shown that colloidal gels formed by networks of aggregated particles can regulate the mechanomorphological features of the gels. In this study, three different types of gelatin based colloidal gels were engineered by modulating the electrostatic-interaction based assembly. Microstructural morphology and mechanics of the colloidal gels were controlled in an independent manner by regulating the mode of particle aggregation and by varying the particle fraction. These highly tunable gels were used to influence endothelial organization during vascular network formation. EC responses on these colloidal gel matrices showed that tenuous strand-like microstructure of colloidal gels favored tubular EC network through increased cell-matrix and cell-cell interactions, while compact dense microstructure inhibited extended EC networks. Increasing gel elasticity for a given microstructure reduced the EC network formation. In fact, with the colloidal gel matrices, we have shown that supportive matrix microstructure can reduce the inhibitory effect of matrix stiffness during EC network formation. This highlighted a bidirectional signaling mechanism from the matrix morphology and stiffness. Overall, this approach presents a strategy to develop gels where the morphology and the mechanics of matrices are segregated, which allows for their distinct roles during EC organization to be interrogated. Conceivably, these colloidal gels with tunable mechanomorphology are highly adaptable materials to regulate vascularization in regenerative tissue engineering. In future, complete understanding of the gelation mechanism with respect to mechanomorphology and underlining the structure-function correlations for endothelial morphogenesis will provide better insight for cell-matrix interactions.

## Materials and Methods

### Materials

Gelatin A (from porcine skin, 300 Bloom, isoelectric point (IEP) = 9) and gelatin B (from bovine skin, 225 Bloom, IEP = 5), glutaraldehyde (GA), sodium chloride, Na-salt of polyacrylic acid (MW: 15,000), glycine, were purchased from Sigma-Aldrich. Other chemicals were purchased at Sigma- Aldrich and used as received unless otherwise noted. Deionized dd-water was used to prepare all the solutions.

### Preparation of colloidal gelatin particles and gels

Colloidal gelatin particles were prepared by a one-step desolvation method. A gelatin solution (10%w/v) was obtained by dissolving gelatin in distilled water under constant heating at 50 °C. After cooling to 35 °C and adjusting the pH to 2.5 (Hydrochloric Acid, 0.1 M) and 10 (Sodium Hydroxide, 0.1 M) for Gelatin A (GelA) and gelatin B (GelB) respectively, acetone was added dropwise to precipitate the particles. Subsequently Glutaraldehyde (GA 8 w/v%) was added to the nano particle suspension. After crosslinking for 20 hours glycine solution (0.75% w/v)) was added to the suspension to neutralize the unreacted aldehyde groups from GA. The suspension was then subjected to 3 cycles of ultracentrifugation (13200 rpm for 5 min). Centrifuged colloidal gelatin particles were used immediately after preparation (to prevent any loss of structure) to aggregate and form gels. Three different colloidal gels were formed by adding electrolyte (4 M sodium chloride - N-colloidal gel), polyelectrolyte (10 w/v% Na-salt of polyacrylic acid - P-colloidal gel) and negatively charged gelatin B particles (AB-colloidal gel). Particle fraction was between 0.05 to 0.2 (w/v) in 2 ml Eppendorf tubes to form these gels. Particle fractions are based on the weight of wet particles (immediately after preparation) to preserve the size and water content of gelatin particles. 0.2 fraction gels were prepared by adding 1 ml of sodium chloride (4 M) and Na-salt of polyacrylic acid (10% w/v in dd-water) to 0.2 gm of gelatin A particles forming N- and P- colloidal gels. AB- gel was prepared by prepared by mixing equal weight fraction of GelA and GelB particles (0.2 w/v). The 0.2 particle fraction samples of the gels were then serially diluted to 0.1 and 0.05 in their respective solvents. To characterize the aggregation, concentration of sodium chloride and Na-salt of polyacrylic acid was varied for N-gel and P-gel respectively, and the ratio of GelA to GelB was varied for AB-gel.

### Characterization of colloidal gelatin particles and their aggregation

Colloidal gelatin particles were imaged from scanning electron microscopy (SEM) and fluorescence imaging (due to auto-fluorescence from glutaraldehyde in FITC (Ex ~ 494 nm/Em ~ Max 520 nm) channel^86^). SEM was performed on air dried sample on glass coverslip with an accelerating voltage of 5.0 kV (Hitachi SU70 FESEM). The samples were coated with 10 nm film of carbon in a high vacuum evaporator for 30 minutes. Fluorescence imaging was performed in confocal microscope (Zeiss LSM-510) at 10x to visualize dispersed particles. Dynamic light scattering (DLS, Zetasizer Nano-S, Malvern Instruments Ltd.) was used to measure the particle size and zeta (ξ) potential by dispersing gelatin particles GelA, GelB in deionized water at a solid content of 0.01 w/v% (measuring time 60 min, one measurement per minute). To monitor the effect of ionic strength on surface charge the particles were dispersed in HEPES buffer with increasing molarity (1 mM, 20 mM, 40 mM, 100 mM). The shift in the charge with increasing pH (3,7,9,) at a set concentration of 1 mM was also measured. DLS measurements were used to characterize the aggregation of gels by measuring size and zeta potential with different concentration sodium chloride (1 M, 2 M, and 4 M) and Na-salt of polyacrylic acid (2.5%, 5% and 10% w/v) for N- and P- respectively, and the ratio of GelA to GelB (4:1, 2:1, and 1:1) was varied for AB-gel. Confocal fluorescence imaging was performed to image the morphology of the aggregates followed creating distance map images using ImageJ (NIH, USA) from the binary images. The map is derived from the original image where every pixel in the background is assigned the shortest possible distance to a foreground pixel using Euclidean distance map algorithm. This allows assessing the organizational map of particle distribution in each aggregate. Quantification of individual aggregates were done calculating equivalent diameter (diameter of the circular projection of an aggregate) and circularity index $(\tfrac{4\pi Area}{Perimete{r}^{2}})$, with index closer to 1 indicates circular structures. For these measurements, individual aggregates were manually traced and quantified using ImageJ from randomly selected fluorescent images for three gels with at least 30 aggregates for each gel type. Data is presented as box plot with box representing 80 percent data distribution with maximum and minimum limit.

### Turbidimetric analysis of colloidal aggregation

Turbidity of the colloidal dispersion and aggregates were measured from the absorbance (optical density) of the samples in UV-vis spectrophotometer (UV1600PC) at room temperature. To calculate the ‘dispersibility factor (*n*)’ optical density (OD) of the samples were measured at wavelength (λ) 650, 600, 550, 500, and 450 nm; and *n* is calculated from the wavelength dependence of OD from the slope of log(OD) vs. log (λ) as,$\,n=-\,\tfrac{d\,\mathrm{log}\,OD}{d\,\mathrm{log}\,\lambda }$^43,44^. Aggregation profile of colloidal particles were obtained by plotting dispersibility factor (*n*) with respect to particle fraction for a given sample. Kinetics of particle aggregation was investigated by measuring the OD with respect to time for 150 minutes for each sample at 650 nm at room temperature.*τ* for all the three aggregates were obtained by fitting Kohlrausch-William-Watts (KWW) function for the decay period function from 5 independent kinetic measurements and data is presented as average ± S.D.

### Morphology of colloidal gel

Gel morphology was characterized SEM and confocal fluorescence imaging (imaged at 20X with z-stacks over 4 µm thickness at interval of 0.1 µm), as described earlier. Gel microstructure was quantified using the Image J software. Binary images were formed by the software by thresholding the original images. After thresholding, Diameter J I-180, a free open source plugin was used to quantify the characteristic length, intersection density, and void space. Diameter J uses axial algorithm which determine centerline of the network strands allowing calculation characteristic network length (in between two intersections) and intersection density (with respect to area) and void space distribution pixel in binary images. For each gel, at more than 50 networks in each gels were analyzed from randomly selected and data is presented as average ± S.D. For confocal images, 3D interactive surface plots from ImageJ was used to generate the heat-map to show the gel morphology and distribution of voids.

### Rheological Characterizations

The viscoelastic properties of colloidal gels were characterized using a rheometer (Bohlin CVOD 100NF). All measurements were performed using a flat steel parallel plate geometry at 25 °C with a gap distance of 200 µm. Oscillatory strain sweeps were performed at variable amplitudes in a strain-controlled mode in the range of 0.01 to 10 while keeping frequency at a constant value of 1 Hz. For selected samples, amplitude sweeps were performed in presence of cell culture media and at 37 °C. These tests determined the storage (elastic) modulus (G′) and the loss (viscous) modulus (G″) and crossover between two moduli. Storage (elastic) moduli (G′) and tan(δ) of three gels with different particle fractions were assessed from linear viscoelastic region of their respective amplitude sweep. Viscosity was analyzed by subjecting it to a controlled shear rate (1/s) in the range of 0.01 to 10 for assessing the shear thinning response. Viscometric data was fitted to power law model to determine power law index (n) and consistency (K). Time-dependent creep response was performed wherein a constant force (shear stress) of 2 Pa (within linear viscoelastic region) was applied to the sample for 100 s and time-dependent formation (strain) was measured for each gel. All rheological experiments were performed 3 times each from 3 different samples, and data for elastic moduli (G′) and tan(δ) is presented as average ± S.D.

### Endothelial cell culture and preparation of cell embedded colloidal gel

Human umbilical vein endothelial cells (HUVEC) were used as endothelial cells in this study. HUVEC were purchased from commercial source (Promocell) and grown in endothelial cell growth medium without VEGF (Cell applications #2110-500). Cells were cultured and maintained in 5% CO_2_ at 37 °C with media change every second day and passaged at 80% confluence. Cells were typically used between passage 4-7 for the experiments. To prepare EC embedded colloidal gels, freshly prepared gels were used after washing five times with phosphate buffered saline (PBS), and finally, one time with endothelial cell media. EC were seeded onto the colloidal gels at a density of 66,600 cells per mg of gel (typically 2 × 10^6^ cells with the gels 0.03 g) by mixing the cells (in 50 µl media) with the colloidal aggregates and cell-gel aggregates were allowed settle in non-adherent U-bottom 96 well plate. After which 100 µl of defined endothelial cell media was added into each of the well. Cell-gel aggregates were maintained in the incubator at 37 °C for characterization at defined time points. Viability of the cells were measured by quantifying total DNA content using PicoGreen assay, according to manufacturers’ instruction, with cell only aggregates as control.

### Analysis of endothelial cell network

To evaluate the endothelial cell networks formed within the three colloidal gels, the gel-EC constructs were grown at 37 °C for 48 hours. Following this, the constructs are fixed overnight in 10% formaldehyde (w/v in dd-water) and subsequently washed twice with PBS. The fixed constructs were embedded in 3% agarose gels and allowed to solidify at 4 °C. Agarose embedded samples were sectioned at 1 µm interval followed by staining with Hematoxylin and Eosin (H&E) using standard protocol in UB Histology core. H&E stained slides were imaged at 40x using Nikon Eclipse Ti-U microscope. Endothelial networks were quantified using ImageJ where the images were transformed into binary images following which skeletonization of the images were done. From these skeletonized images, the length of the tubular networks, and the three-point junctions per network were calculated using ImageJ. Images were acquired from randomly selected slides from different sections of each sample. Tube lengths and number of branches per tube are analyzed from more than 50 individual EC networks in each sample. Tube length data is presented as box plot with box representing 80 percent data distribution with maximum and minimum limit and number of branches per tube data is presented as average ± S.D.

### Western blot analysis

Immunoblots were adapted from an established protocol^87^. Proteins were extracted by the addition of trichloracetic acid (TCA) buffer containing 10% TCA; 10 mM Tris-HCl pH 8; 25 mM ammonium acetate, and 1 mM EDTA. Acid-washed glass beads were added to the scaffold and cell pellet mixture. Cells were lysed by five consecutive one-minute vortex pulses with one-minute rests on ice using fast prep multi-vortex (Labline instrument, Melrose, IL). Proteins were precipitated by centrifugation at 4 °C at 16000 g for 10 min. Protein pellets were thoroughly re-suspended using resuspension buffer containing 0.1 M Tris-HCl pH 11 and 3% SDS by boiling the suspension for 5 min at 95 °C. Total protein concentration was measured using Biorad BCA protein assay kit (Pierce™ Microplate BCA Protein Assay Kit catalog # 23252). Equal amounts of 4X laemmli sample buffer was added to the re-suspended protein solution and boiled for 5 min at 95 °C. Extracted proteins were loaded onto 10% SDS-PAGE separating gel. Approximately 10 μg of protein was loaded per lane. Proteins were transferred to nitrocellulose membranes (protran BA85, VWR International Inc. Bridgeport NJ). Membrane was blocked either with 5% nonfat milk or 5% BSA in 10 mM Tris-HCl pH8, 150 mM NaCl and 0.05% Tween 20. Immunoblots were visualized using Western Bright ECL HRP substrate (Menlo Park, CA; LPS #K-12045-D20). To detect Focal adhesion kinase (FAK), primary antibody (FAK antibody # 3285) was used at 1: 1000 dilution, to detect phosphor-FAK primary antibody [Phospho-FAK (Tyr397) Antibody #3283] was used at 1:1000 dilution, and to detect VE-cadherin, primary antibody (VE-cadherin antibody # 2158) was used at 1: 1000 dilution, according to manufacturers’ protocol with the corresponding secondary antibody. For all western blot analysis, β-actin [β-Actin (13E5) Rabbit mAb #4970] was used as control. All primary and secondary antibodies were purchased from Cell Signaling Technology, MA, US. Quantification of western blots was performed from densitometric analysis of bands using Image Lab 3.0, Biorad software and bands were normalized with respect to β-actin band. Measurements were performed at least 3 times and data is presented as average ± S.D.

### Data analysis

Data is presented either as an average with ± standard deviation (error bar representing standard deviation) or as a box-plot with 80% data points embedded in the box (error bar representing maximum and minimum value). Statistical significance was determined by multiple comparison ANOVA followed Tukey’s test. P value ≤ 0.05 is designated with * and value ≤ 0.01 is designated with **. P value ≤ 0.05 is considered significant.

## Supplementary information

Supplementary Information

## Data Availability

All data generated or analyzed during this study are included in this published article and its supplementary information files.

## References

[1] Lu PJ, Weitz DA (2013). Colloidal Particles: Crystals, Glasses, and Gels. Annual Review of Condensed Matter Physics.

[2] Saunders BR, Vincent B (1999). Microgel particles as model colloids: theory, properties and applications. Advances in Colloid and Interface Science.

[3] Ikeda S, Zhong Q (2012). Polymer and Colloidal Models Describing Structure-Function Relationships. Annual Review of Food Science and Technology.

[4] Lu, P. J. *et al*. Gelation of particles with short-range attraction. *Nature* **453**, 499, 10.1038/nature06931, https://www.nature.com/articles/nature06931#supplementary-information (2008). 10.1038/nature06931 18497820

[5] Joshi YM (2014). Dynamics of Colloidal Glasses and Gels. Annual Review of Chemical and Biomolecular Engineering.

[6] Sutherland DN (1967). A theoretical model of floc structure. Journal of Colloid and Interface Science.

[7] Lazzari S, Nicoud L, Jaquet B, Lattuada M, Morbidelli M (2016). Fractal-like structures in colloid science. Advances in Colloid and Interface Science.

[8] Lu PJ, Conrad JC, Wyss HM, Schofield AB, Weitz DA (2006). Fluids of Clusters in Attractive Colloids. Physical Review Letters.

[9] Prasad V (2003). Rideal Lecture Universal features of the fluid to solid transition for attractive colloidal particles. Faraday Discussions.

[10] Trappe V, Prasad V, Cipelletti L, Segre PN, Weitz DA (2001). Jamming phase diagram for attractive particles. Nature.

[11] Kniazeva E, Putnam AJ (2009). Endothelial cell traction and ECM density influence both capillary morphogenesis and maintenance in 3-D. Am J Physiol-Cell Ph.

[12] Moon JJ (2010). Biomimetic hydrogels with pro-angiogenic properties. Biomaterials.

[13] Seliktar D (2004). MMP-2 sensitive, VEGF-bearing bioactive hydrogels for promotion of vascular healing. Journal of Biomedical Materials Research Part A.

[14] Silva EA, Kim E-S, Kong HJ, Mooney DJ (2008). Material-based deployment enhances efficacy of endothelial progenitor cells. Proceedings of the National Academy of Sciences.

[15] Hanjaya-Putra D (2011). Controlled activation of morphogenesis to generate a functional human microvasculature in a synthetic matrix. Blood.

[16] Lesman A (2011). Engineering vessel-like networks within multicellular fibrin-based constructs. Biomaterials.

[17] Davis GE, Saunders WB (2006). Molecular Balance of Capillary Tube Formation versus Regression in Wound Repair: Role of Matrix Metalloproteinases and Their Inhibitors. Journal of Investigative Dermatology Symposium Proceedings.

[18] Duffy GP (2011). Towards *in vitro* vascularisation of collagen-GAG scaffolds. Eur Cell Mater.

[19] Wolf K (2013). Physical limits of cell migration: Control by ECM space and nuclear deformation and tuning by proteolysis and traction force. The Journal of Cell Biology.

[20] Ghajar CM (2008). The Effect of Matrix Density on the Regulation of 3-D Capillary Morphogenesis. Biophysical Journal.

[21] Pathak A, Kumar S (2011). Biophysical regulation of tumor cell invasion: moving beyond matrix stiffness. Integrative Biology.

[22] Huanan W (2011). Oppositely Charged Gelatin Nanospheres as Building Blocks for Injectable and Biodegradable Gels. Advanced Materials.

[23] Wang M, He L, Yin Y (2013). Magnetic field guided colloidal assembly. Materials Today.

[24] Sánchez-Iglesias A (2012). Hydrophobic Interactions Modulate Self-Assembly of Nanoparticles. ACS Nano.

[25] Tohver V, Chan A, Sakurada O, Lewis JA (2001). Nanoparticle Engineering of Complex Fluid Behavior. Langmuir.

[26] Wang Y (2012). Colloids with valence and specific directional bonding. Nature.

[27] Bishop KJ, Wilmer CE, Soh S, Grzybowski BA (2009). Nanoscale Forces and Their Uses in Self-Assembly. Small.

[28] Lin MY (1990). Universal reaction-limited colloid aggregation. Physical Review A.

[29] Lin MY (1990). Universal diffusion-limited colloid aggregation. Journal of Physics: Condensed Matter.

[30] Lin MY (1989). Universality in colloid aggregation. Nature.

[31] Wang Q, Wang L, Detamore MS, Berkland C (2008). Biodegradable Colloidal Gels as Moldable Tissue Engineering Scaffolds. Advanced Materials.

[32] Van Tomme SR, van Steenbergen MJ, De Smedt SC, van Nostrum CF, Hennink WE (2005). Self-gelling hydrogels based on oppositely charged dextran microspheres. Biomaterials.

[33] Davidenko N (2016). Evaluation of cell binding to collagen and gelatin: a study of theeffect of 2D and 3D architecture and surface chemistry. Journal of Materials Science: Materials in Medicine.

[34] Van den Steen PE (2002). Biochemistry and Molecular Biology of Gelatinase B or Matrix Metalloproteinase-9 (MMP-9). Critical Reviews in Biochemistry and Molecular Biology.

[35] Ethirajan A, Schoeller K, Musyanovych A, Ziener U, Landfester K (2008). Synthesis and Optimization of Gelatin Nanoparticles Using the Miniemulsion Process. Biomacromolecules.

[36] Kommareddy S, Amiji MM (2008). Preparation and Loading of Gelatin Nanoparticles. Cold Spring Harbor Protocols.

[37] Azarmi S (2006). Optimization of a two-step desolvation method for preparing gelatin nanoparticles and cell uptake studies in 143B osteosarcoma cancer cells. J Pharm Pharm Sci.

[38] Oncsik T, Trefalt G, Csendes Z, Szilagyi I, Borkovec M (2014). Aggregation of Negatively Charged Colloidal Particles in the Presence of Multivalent Cations. Langmuir.

[39] Trefalt G, Szilagyi I, Borkovec M (2013). Poisson–Boltzmann description of interaction forces and aggregation rates involving charged colloidal particles in asymmetric electrolytes. Journal of Colloid and Interface Science.

[40] Jiang J, Oberdörster G, Biswas P (2009). Characterization of size, surface charge, and agglomeration state of nanoparticle dispersions for toxicological studies. Journal of Nanoparticle Research.

[41] Brunel F, Pochard I, Turesson M, Gauffinet S, Labbez C (2017). Elastic Response of Cementitious Gels to Polycation Addition. ACS Omega.

[42] Pickrahn K, Rajaram B, Mohraz A (2010). Relationship between Microstructure, Dynamics, and Rheology in Polymer-Bridging Colloidal Gels. Langmuir.

[43] Long JA, Osmond DWJ, Vincent B (1973). The equilibrium aspects of weak flocculation. Journal of Colloid and Interface Science.

[44] Snowden, M. J., Gracia, L. H. & Nur, H. In *New frontiers in colloid science* 148–165 (2008).

[45] Gregory J (2009). Monitoring particle aggregation processes. Advances in Colloid and Interface Science.

[46] Iselau F (2016). Formation and relaxation kinetics of starch–particle complexes. Soft Matter.

[47] Bizmark N, Ioannidis MA (2015). Effects of Ionic Strength on the Colloidal Stability and Interfacial Assembly of Hydrophobic Ethyl Cellulose Nanoparticles. Langmuir.

[48] French RA (2009). Influence of Ionic Strength, pH, and Cation Valence on Aggregation Kinetics of Titanium Dioxide Nanoparticles. Environmental Science & Technology.

[49] Brunel F, Pochard I, Gauffinet S, Turesson M, Labbez C (2016). Structure and Yielding of Colloidal Silica Gels Varying the Range of Interparticle Interactions. The Journal of Physical Chemistry B.

[50] Stoll S, Buffle J (1996). Computer Simulation of Bridging Flocculation Processes: The Role of Colloid to Polymer Concentration Ratio on Aggregation Kinetics. Journal of Colloid and Interface Science.

[51] Varadan P, Solomon MJ (2001). Shear-Induced Microstructural Evolution of a Thermoreversible Colloidal Gel. Langmuir.

[52] Arzumand A, Srinivas S, Yuan Y, Zhou C, Sarkar D (2016). Mechano-Morphological Characterization of Polyethylene-Glycol Based Polyurethane Microgel. Macromolecular Materials and Engineering.

[53] Thomas JJ, Jennings HM (2006). A colloidal interpretation of chemical aging of the C-S-H gel and its effects on the properties of cement paste. Cement and Concrete Research.

[54] Cipelletti L, Manley S, Ball RC, Weitz DA (2000). Universal Aging Features in the Restructuring of Fractal Colloidal Gels. Physical Review Letters.

[55] Sprakel J, Lindström SB, Kodger TE, Weitz DA (2011). Stress Enhancement in the Delayed Yielding of Colloidal Gels. Physical Review Letters.

[56] Dinsmore AD, Prasad V, Wong IY, Weitz DA (2006). Microscopic Structure and Elasticity of Weakly Aggregated Colloidal Gels. Physical Review Letters.

[57] Shih W-H, Shih WY, Kim S-I, Liu J, Aksay IA (1990). Scaling behavior of the elastic properties of colloidal gels. Physical Review A.

[58] Wu H, Morbidelli M (2001). A Model Relating Structure of Colloidal Gels to Their Elastic Properties. Langmuir.

[59] Mani D, Huanan W, E. KT, Shima P, G. LSC (2017). Highly Elastic and Self-Healing Composite Colloidal Gels. Advanced Materials.

[60] Wang H (2012). Comparison of micro- vs. nanostructured colloidal gelatin gels for sustained delivery of osteogenic proteins: Bone morphogenetic protein-2 and alkaline phosphatase. Biomaterials.

[61] Ayesha A, Shruti S, Yuan Y, Chi Z, Debanjan S (2016). Mechano-Morphological Characterization of Polyethylene-Glycol Based Polyurethane Microgel. Macromolecular Materials and Engineering.

[62] Lindström SB, Kodger TE, Sprakel J, Weitz DA (2012). Structures, stresses, and fluctuations in the delayed failure of colloidal gels. Soft Matter.

[63] Gopalakrishnan V, Zukoski CF (2007). Delayed flow in thermo-reversible colloidal gels. Journal of Rheology.

[64] Landrum BJ, Russel WB, Zia RN (2016). Delayed yield in colloidal gels: Creep, flow, and re-entrant solid regimes. Journal of Rheology.

[65] Ewoldt RH, Creep GHM (2007). ringing in rheometry or how to deal with oft-discarded data in step stress tests!. Rheology Bulletin.

[66] Jaishankar, A. & McKinley, G. H. Power-law rheology in the bulk and at the interface: quasi-properties and fractional constitutive equations. *Proceedings of the Royal Society A: Mathematical, Physical and Engineering Science* **469**, 10.1098/rspa.2012.0284 (2013).

[67] Goudoulas TB, Germann N (2016). Viscoelastic properties of polyacrylamide solutions from creep ringing data. Journal of Rheology.

[68] Jaishankar A, Sharma V, McKinley GH (2011). Interfacial viscoelasticity, yielding and creep ringing of globular protein–surfactant mixtures. Soft Matter.

[69] Schweller RM, West JL (2015). Encoding Hydrogel Mechanics via Network Cross-Linking Structure. ACS Biomaterials Science & Engineering.

[70] Chwalek, K., Tsurkan, M. V., Freudenberg, U. & Werner, C. Glycosaminoglycan-based hydrogels to modulate heterocellular communication in *in vitro* angiogenesis models. *Scientific Reports* **4**, 4414, 10.1038/srep04414https://www.nature.com/articles/srep04414#supplementary-information (2014). 10.1038/srep04414 PMC3958722 24643064

[71] Bott K (2010). The effect of matrix characteristics on fibroblast proliferation in 3D gels. Biomaterials.

[72] Lou J, Stowers R, Nam S, Xia Y, Chaudhuri O (2018). Stress relaxing hyaluronic acid-collagen hydrogels promote cell spreading, fiber remodeling, and focal adhesion formation in 3D cell culture. Biomaterials.

[73] Van Goethem E, Poincloux R, Gauffre F, Maridonneau-Parini I, Le Cabec V (2010). Matrix Architecture Dictates Three-Dimensional Migration Modes of Human Macrophages: Differential Involvement of Proteases and Podosome-Like Structures. The Journal of Immunology.

[74] Vernon RB, Angello JC, Iruela-Arispe ML, Lane TF, Sage EH (1992). Reorganization of basement membrane matrices by cellular traction promotes the formation of cellular networks *in vitro*. Lab Invest.

[75] Sacharidou A (2010). Endothelial lumen signaling complexes control 3D matrix–specific tubulogenesis through interdependent Cdc42- and MT1-MMP–mediated events. Blood.

[76] Douglas AM (2017). Dynamic assembly of ultrasoft colloidal networks enables cell invasion within restrictive fibrillar polymers. Proceedings of the National Academy of Sciences.

[77] Mui KL, Chen CS, Assoian RK (2016). The mechanical regulation of integrin–cadherin crosstalk organizes cells, signaling and forces. Journal of Cell Science.

[78] Braren R (2006). Endothelial FAK is essential for vascular network stability, cell survival, and lamellipodial formation. The Journal of Cell Biology.

[79] Ilic D (2003). Focal adhesion kinase is required for blood vessel morphogenesis. Circ Res.

[80] Gory-Faure S (1999). Role of vascular endothelial-cadherin in vascular morphogenesis. Development.

[81] Haskell H (2003). Focal Adhesion Kinase Is Expressed in the Angiogenic Blood Vessels of Malignant Astrocytic Tumors *in Vivo* and Promotes Capillary Tube Formation of Brain Microvascular Endothelial Cells. Clinical Cancer Research.

[82] Bach TL (1998). VE-Cadherin Mediates Endothelial Cell Capillary Tube Formation in Fibrin and Collagen Gels1. Experimental Cell Research.

[83] Seong J (2013). Distinct biophysical mechanisms of focal adhesion kinase mechanoactivation by different extracellular matrix proteins. Proceedings of the National Academy of Sciences.

[84] Pompe T (2009). Dissecting the Impact of Matrix Anchorage and Elasticity in Cell Adhesion. Biophysical Journal.

[85] Giannone G, Sheetz MP (2006). Substrate rigidity and force define form through tyrosine phosphatase and kinase pathways. Trends in Cell Biology.

[86] Collins JS, Goldsmith TH (1981). Spectral properties of fluorescence induced by glutaraldehyde fixation. Journal of Histochemistry & Cytochemistry.

[87] Basu S (2016). Spatial landmarks regulate a Cdc42-dependent MAPK pathway to control differentiation and the response to positional compromise. Proceedings of the National Academy of Sciences.

