# Supplementary material for: Colloidal Gels with Tunable Mechanomorphology Regulate Endothelial Morphogenesis
Source: Sci Rep. 2019 Jan 31;9:1072. doi: 10.1038/s41598-018-37788-w (PMC6355882; doi:10.1038/s41598-018-37788-w)
Supplement: Supplementary file 1 — Supplementary Information [file 41598_2018_37788_MOESM1_ESM.pdf]

Supplementary Information for:

Colloidal Gels with Tunable Mechanomorphology Regulate Endothelial Morphogenesis

Smruti K. Nair[a], Sukanya Basu[b], Ballari Sen[a], Meng-Hsuan Lin[a], Arati N. Kumar[a], Yuan Yuan[a], Paul J. Cullen[b], Debanjan Sarkar[a,c,*]

[a]Department of Biomedical Engineering, University at Buffalo, The State University of New York, Buffalo, NY 14260, USA

[b]Department of Biological Sciences, University at Buffalo, The State University of New York, Buffalo, NY 14260, USA

[c]Department of Chemical and Biological Engineering, University at Buffalo, The State University of New York, Buffalo, NY 14260, USA

*Correspondence to: D. Sarkar.
Biomedical Engineering, University at Buffalo, Ph: 716-645-8497, Fax: 716-645-2207
Email: debanjan@buffalo.edu

Supplementary Figures:

a)

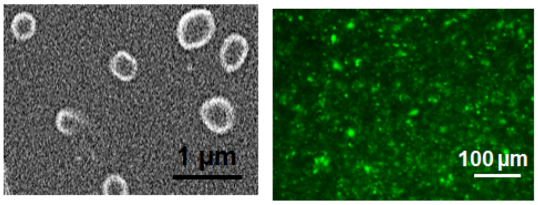

Size (nm)	Zeta Potential(mV)
358.7 $\pm$ 3.7	-24.1 $\pm$ 0.2

b)

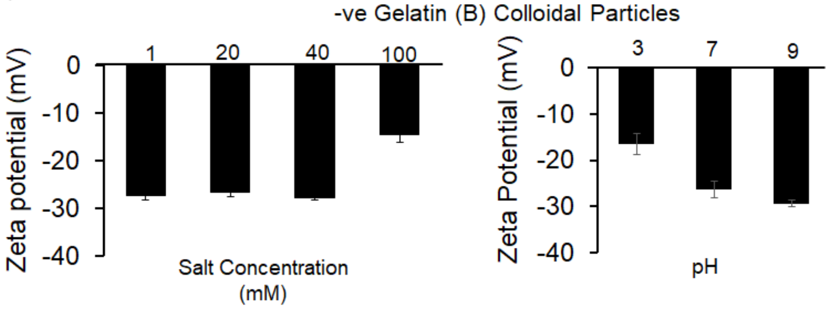

Figure S1. a) Microscopic images of colloidal gelatin B particles from scanning electron microscopy and fluorescence images showing submicron particles which are uniformly dispersed. Average size and surface charge (zeta potential) measured by dynamic light scattering. b) Variation of zeta potential of colloidal gelatin B particles as a function of ionic strength (from HEPES buffer at pH 7) and pH (with 1 mM HEPES buffer).

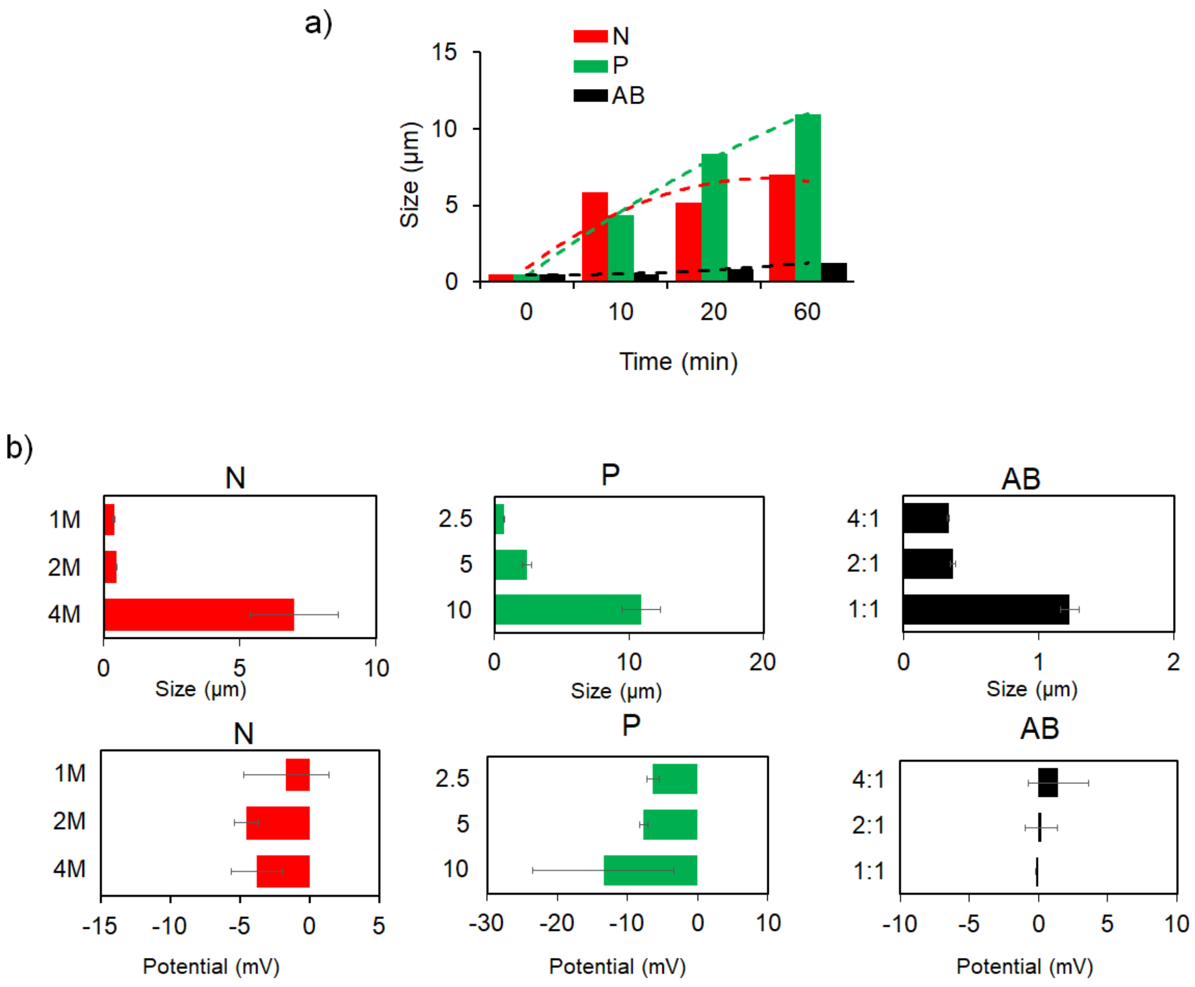

Figure S2. a) Aggregation of gelatin A particle with sodium 4M sodium chloride (N), 10% Na-salt of polyacrylic acid (P), and 1:1 negatively charged gelatin B (AB) particles measured from size of aggregates by DLS with respect to time. b) Variation of size and zeta potential following addition of different concentration of sodium chloride (N), Na-salt of polyacrylic acid (P), and negatively charged gelatin B (AB) particles showing aggregation of particles due to electrostatic interactions.

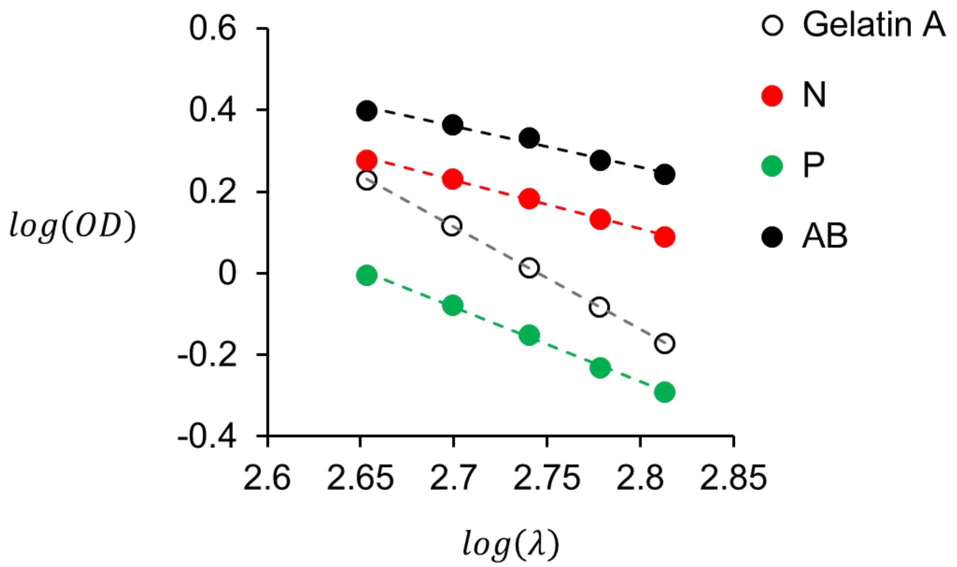

Figure S3. Plot of log (optical density) with respect to log (wave length) for colloidal gelatin A and N-, P- and AB- aggregates to determine the dispersibility factor ‘n’ from the slope. Increased aggregation show reduced negative slope i.e. increase in ‘n’

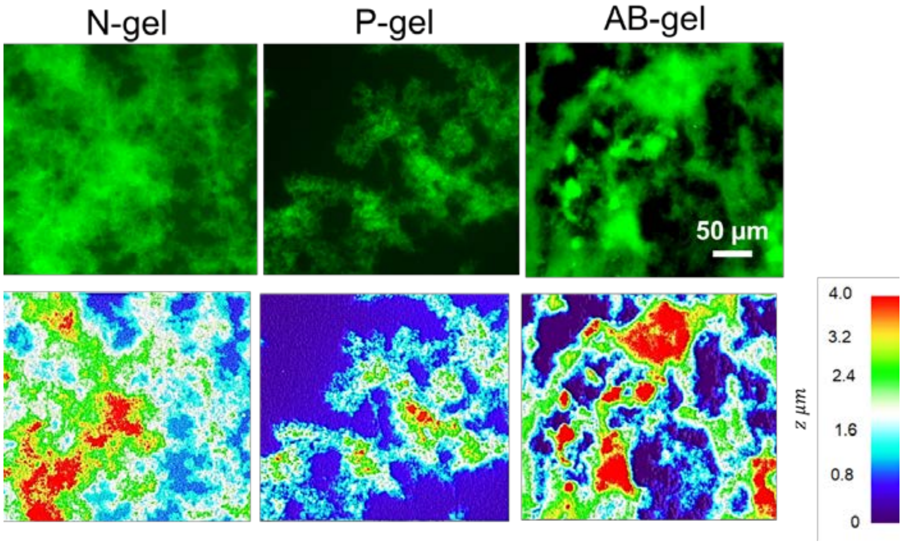

Figure S4. Confocal scanning fluorescent images and 3D interactive surface plot (color code indicates z-depth) of gels showing morphology and spatial distribution of voids in the N-, P- and AB-gel with 0.2 particle fraction.

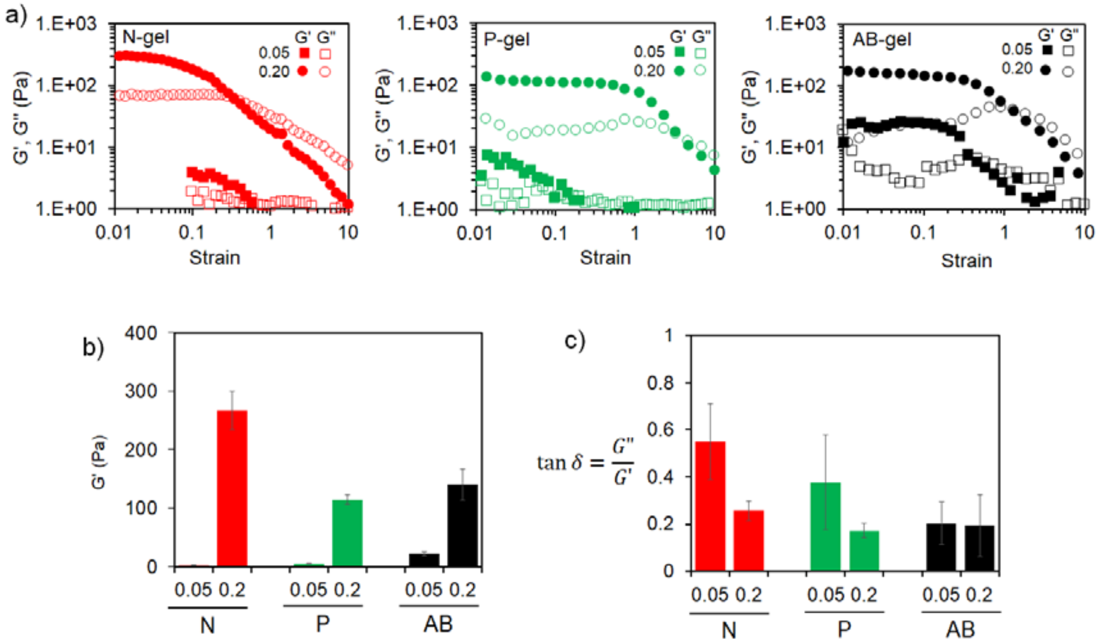

Figure S5. a) Strain amplitude sweep of N-, P-and AB-gel at 0.05 and 0.2 particle fractions measured at constant frequency of 1 Hz in presence of cell culture media and 37°C. b) Variation of elastic moduli (G') and c) tan(δ) of N-, P- and AB-gel at 0.05 and 0.2 particle fractions measured from linear viscoelastic region in presence of cell culture media and 37°C.

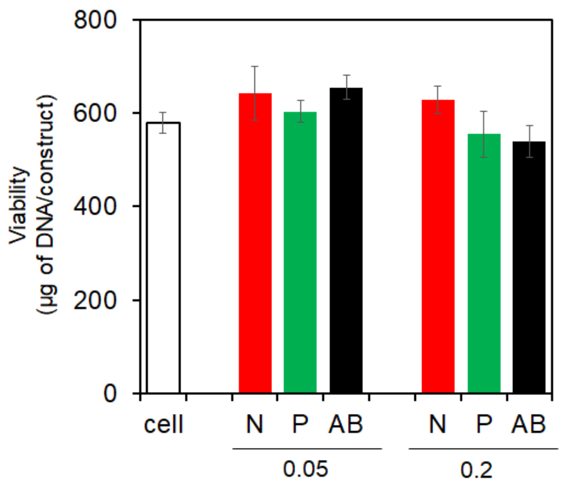

Figure S6. Viability of endothelial cells in the N-, P- and AB-gel with 0.05 and 0.2 particle fraction measured by total DNA content using PicoGreen assay after 48 hrs showing no change in viability.

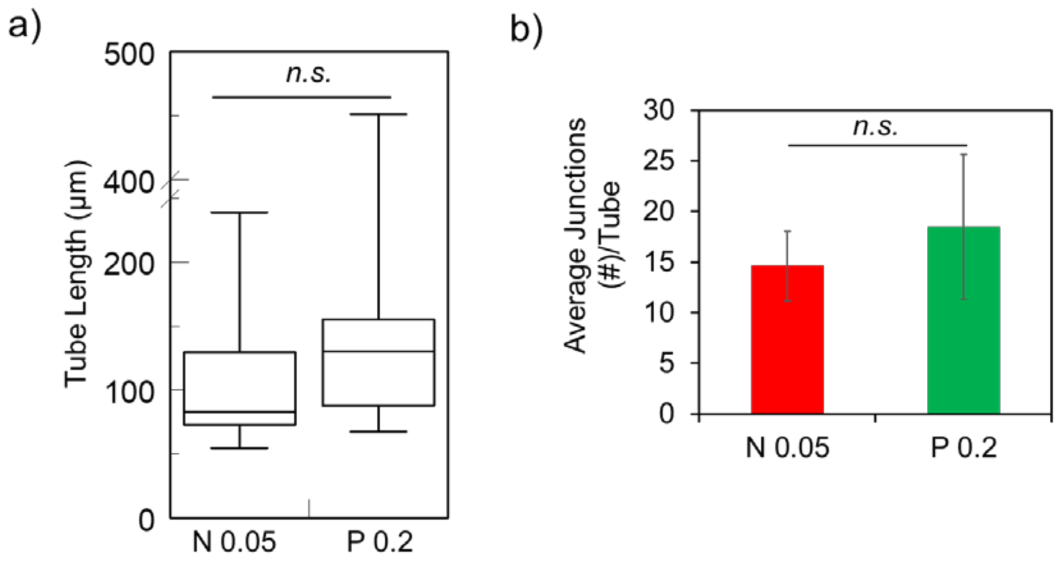

Figure S7. Comparison of EC networks between N-gel at 0.05 particle fraction and P-gel at 0.2 particle fraction from a) from tube length and b) numbers junction per tube (*n.s.* = statistically not significant).

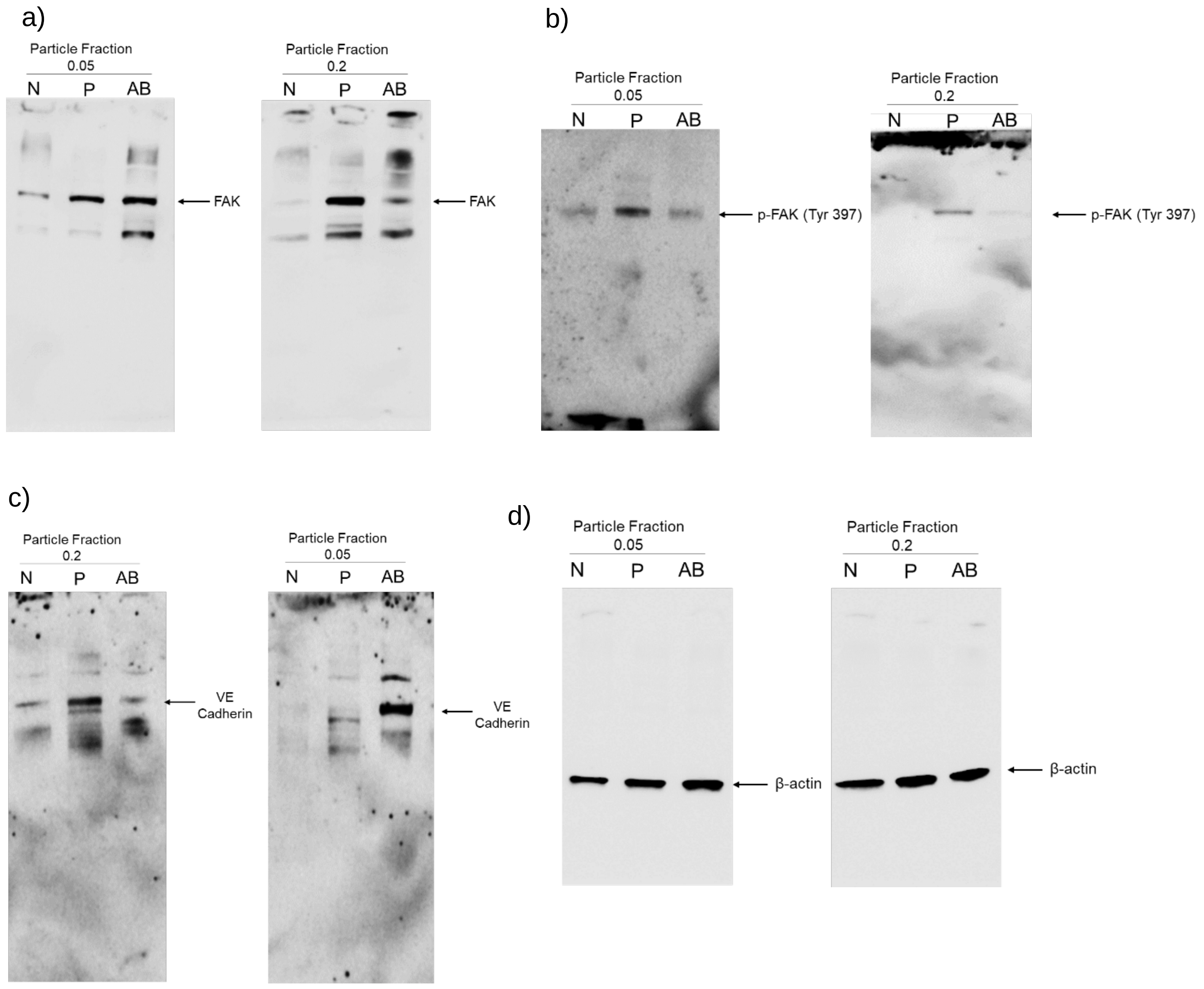

Figure S8. Full length blots corresponding to figure 8 for a) FAK, b) p-FAK, c) VE-cadherin, and d) β-actin. The blots correspond to N-, P-, and AB-gel at particle fraction 0.05 in figure 8a and at particle fraction 0.2 in figure 8c.

Supplementary Table:

Table S1: Power law analysis (i.e., *n* and *K*) from viscometric measurements for N-, P- and AB-gel from different particle fraction

Gel / *Particle Fraction*	N-gel		P-gel		AB-gel	
	Power Law Index (n)	Consistency (K) (Pa.s^n)	Power Law Index (n)	Consistency (K) (Pa.s^n)	Power Law Index (n)	Consistency (K) (Pa.s^n)
0.05	0.18	1.1	0.57	0.6	0.32	24
0.10	0.25	7.8	0.34	0.8	0.29	29
0.20	0.23	22.3	0.32	9.3	0.13	70.2

Power Law:

$$\eta = K(\dot{\gamma})^{n-1}$$

η = viscosity

$\dot{\gamma}$ = shear rate
